# Prevalence and risk factors for postoperative ileus in colorectal cancer patients: a systematic review and meta-analysis

**DOI:** 10.3389/fonc.2025.1742152

**Published:** 2026-01-16

**Authors:** Dongqing Guo, Shanshan Peng, Wenzhuo An, Jiayan Yu, Xin Chu

**Affiliations:** 1 School of Nursing, Chengdu University of Traditional Chinese Medicine, Chengdu, Sichuan, China; 2 Hospital of Chengdu University of Traditional Chinese Medicine, Chengdu, Sichuan, China

**Keywords:** colorectal cancer, meta-analysis, postoperative ileus (POI), prevalence, risk factors

## Abstract

**Background:**

Postoperative ileus (POI) is a common complication following colorectal cancer surgery, with its incidence and risk factors remaining controversial. This study aims to investigate the overall prevalence of POI in colorectal cancer patients and its risk factors through a meta-analysis.

**Methods:**

A systematic search was conducted in the Web of Science, Cochrane Library, PubMed, and Embase databases from their inception to August 7, 2025. Two researchers independently screened studies, extracted data, and assessed the risk of bias in the included studies. Statistical analysis was performed using Stata 15.1.

**Results:**

Thirty studies involving 73,433 patients were included, with 7,463 cases of POI. The overall Prevalence of POI in colorectal cancer patients was [ES = 9%, 95% CI: 8%–11%]. Meta-analysis showed that male [OR = 2.20, 95% CI: 1.76–2.76], operative time >3 hours [OR = 1.75, 95% CI: 1.20–2.40], open surgery [OR = 2.95, 95% CI: 2.20–3.95], ileostomy [OR = 4.78, 95% CI: 2.30–9.94], previous abdominal surgery [OR = 2.21, 95% CI: 1.75–2.79], and age≥65 years [OR = 1.04, 95% CI: 1.01–1.06] may be potential risk factors for POI in colorectal cancer patients. The included studies were of high quality, and no significant publication bias was detected.

**Conclusion:**

Based on existing evidence, male, operative time >3 hours, open surgery, ileostomy, previous abdominal surgery, and age≥65 years may be potential risk factors for POI in colorectal cancer patients.

**Systematic review registration:**

https://www.crd.york.ac.uk/prospero/, identifier CRD420251121761.

## 1 Introduction

Colorectal cancer ranks among the most common digestive system malignancies worldwide, occupying third place in incidence and fifth place in mortality among all cancers (1). In recent years, the disease burden of colorectal cancer has continued to increase, with a significant upward trend in both new cases and deaths worldwide. According to GLOBOCAN projections, by 2040, the global annual Incidence is expected to rise to approximately 3.2 million new cases, with deaths projected to reach 1.6 million (2).

Currently, radical surgical resection remains the primary curative treatment for colorectal cancer patients. Simultaneously, for specific patient subgroups with mismatch repair deficiency (dMMR) or microsatellite instability-high (MSI-H), immunotherapy offers a potential non-surgical curative treatment option for some individuals (3–5). To further reduce recurrence rates and improve prognosis, a combination of comprehensive therapeutic strategies is often required. Neoadjuvant chemotherapy can shrink the preoperative tumor volume, lower the recurrence rate of micrometastases, and increase the rate of R0 resection (6, 7). Adjuvant chemotherapy is commonly administered to high-risk stage II and stage III patients postoperatively, aiming to eradicate potential micrometastases present at the time of resection and reduce recurrence risk (8). Radiation therapy is employed for locally advanced rectal cancer, serving as adjuvant treatment to reduce recurrence rates and as preoperative therapy to improve local control and achieve tumor downstaging (9).

Despite advances in treatment, postoperative complications remain prevalent, significantly impacting patient recovery, quality of life, and long-term survival outcomes. Among these, postoperative ileus (POI) is a common and clinically significant complication following colorectal surgery. POI is characterized by a transient disturbance of gastrointestinal motility, resulting in delayed recovery of bowel function (10). Clinical manifestations include nausea, vomiting, abdominal distension, and delayed or absent passage of flatus and stool (10). POI not only significantly reduces postoperative comfort but also prolongs hospital stays, increases readmission rates, and elevates the risk of perioperative adverse events, thereby adversely affecting patient prognosis (11). A large-scale study across 160 U.S. hospitals demonstrated that patients with POI experienced significantly longer average hospital stays (11.4 days vs. 5.12 days) (12). Another study indicated that readmission rates for such patients can reach approximately 10% (13). Furthermore, POI can impair fluid, electrolyte, and nutrient absorption, triggering disturbances in the internal environment and compromised immune function (14). This increases the risk of severe complications such as infection, sepsis, and even intestinal ischemia and necrosis (15). Some patients may require secondary surgery or face mortality risks, imposing a heavy burden on both patients and the healthcare system (15, 16). Therefore, early identification of risk factors for POI following colorectal cancer surgery is crucial for implementing targeted prevention and timely intervention.

The pathogenesis of POI is complex, involving neurogenic mechanisms, inflammatory and immune responses, medications, and postoperative stress (17, 18). Its incidence varies considerably across studies, with generally reported rates between 10% and 30%. This variation is potentially related to surgical approach, intraoperative procedures, and patient baseline conditions (19). In recent years, multiple studies have explored potential risk factors for postoperative ileus following colorectal cancer surgery, including age, gender, intraoperative procedures, and medication use. However, results remain inconsistent, lacking unified conclusions. Therefore, this study aims to resolve these controversies by conducting a meta-analysis and systematic review to integrate existing evidence and identify the primary factors influencing postoperative ileus after colorectal cancer surgery. The ultimate goal is to improve the quality of life and prognosis of colorectal cancer patients through the prevention and management of postoperative ileus.

## 2 Materials and methods

This study was conducted and reported in accordance with the Preferred Reporting Items for Systematic Reviews and Meta-Analyses (PRISMA) guidelines (20). In addition, the study protocol was registered on the International Prospective Register of Systematic Reviews. (registration ID: CRD420251121761).

### 2.1 Search strategy

As of August 7, 2025, we systematically searched PubMed, Embase, Web of Science, and the Cochrane Library databases to identify observational studies on the incidence and risk factors of POI in colorectal cancer patients. The search employed a strategy combining subject headings and free-text terms. Search terms included “Colorectal Neoplasms,” “postoperative ileus,” “Postoperative Complications,” and “Risk Factors.” Detailed search strategies are provided in **Supplementary Material 1**.

### 2.2 Inclusion and exclusion criteria

Studies were included if they met the following criteria:

1. The study subjects were patients over 18 years old who underwent elective surgeries for colorectal cancer.
2. The study design type was observational studies, including prospective and retrospective studies.
3. The risk factors and Incidence of postoperative ileus were reported.
4. The OR values of the predictive factors and their 95% confidence intervals were provided, or the above data could be obtained through sufficient data calculation.

The exclusion criteria were as follows:

1. full texts unavailable;
2. insufficient data for analysis;
3. duplicate publications;
4. case reports, editorials, conference abstracts, comments, letters, study protocols, and reviews.

### 2.3 Data extraction

Two reviewers independently screened the titles and abstracts of the retrieved studies, excluding those that were clearly irrelevant. Full-text assessments of the remaining studies were then conducted independently to determine their eligibility. Any discrepancies were resolved through discussion or consultation with a third reviewer. Data were independently extracted using standardized forms: first author, publication year, country, study design, sample size, gender, mean Age, TNM staging, criteria for diagnosing POI, risk factors, and OR with its 95% CI. For key data unavailable, attempts were made to obtain it by contacting the corresponding author. Studies where data remained inaccessible were excluded.

### 2.4 Quality assessment

The Newcastle-Ottawa Scale (NOS) (21) was used to assess the methodological quality of the included observational studies. The NOS evaluates studies across three domains: selection, comparability, and outcome/exposure. It consists of 8 items and has a total score of 9. Studies scoring 7 points or higher were considered high-quality and included. Two reviewers independently conducted the assessments. Any discrepancies were resolved through consensus or by consultation with a third reviewer.

### 2.5 Statistical analysis

Statistical analysis of the data was performed using Stata 15.1. Effect sizes were expressed as odds ratios (ORs) and their 95% confidence intervals (CIs). Heterogeneity was assessed using the Q test combined with the I^2^ statistic. A fixed-effects model was used when P > 0.1 and I^2^ < 50%; a random-effects model was used when P ≤ 0.1 or I^2^ ≥ 50%. Sensitivity analyses were conducted for studies with high heterogeneity. Publication bias was assessed using funnel plots and Egger’s test. If Egger’s test yielded P < 0.05, indicating publication bias, the trim-and-fill method was applied to validate the stability.

## 3 Results

### 3.1 Study selection

**Figure 1:**
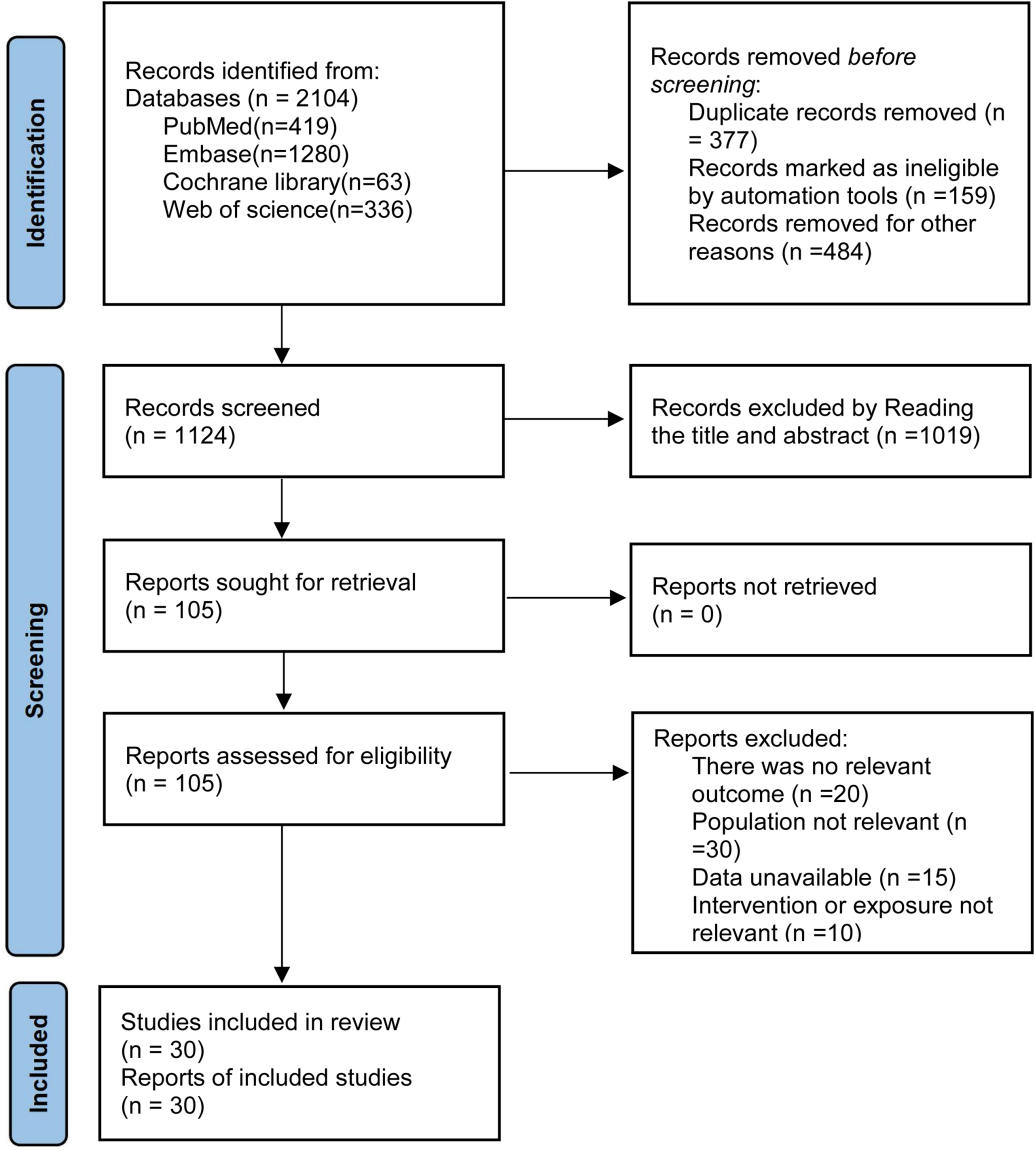
PRISMA flow diagram of the study selection process.

A total of 2,104 records were retrieved through database searches. After excluding 377 duplicate publications, the titles and abstracts of 1,727 studies were screened, and 105 studies met the potential inclusion criteria. Full-text review excluded 75 studies that failed to meet inclusion criteria, primarily due to: irrelevant outcomes (n=20), incompatible study population (n=30), unavailable data (n=15), and exposure factors not relevant to the research focus (n=10). Ultimately, 30 studies (14, 22–50) were included in the quantitative synthesis. The literature screening process is illustrated in **Figure 1**.

### 3.2 Study characteristic

A total of 30 studies were included in this meta-analysis, comprising 29 cohort studies (14, 22–28, 30–50) and one case-control study (29), involving 73,433 patients with colorectal cancer. Among them, 7,463 patients developed POI. These studies were published between 2008 and 2025. Males accounted for 53.46% of participants, while females constituted 46.56%. The mean age range was 52.31 to 73.60 years. Detailed characteristics of all included studies are presented in **Table 1**.

**Table 1:** Characteristics of included studies.

Study	Year	Study design	Country	Sample size	Number of POI	Gender (M/F)	TNM	Mean age	POI diagnostic criteria	Regression model
Campana, J. (23)	2017	cohort study	Argentina	547	37	265/282	I-IV	68.5	Clinical symptoms	logistic regression
Eto, K. (30)	2018	cohort study	Japan	849	37	536/313	0-IV	68	Both clinical symptoms and imaging findings	logistic regression
Garfinkle, R. (24)	2020	cohort study	Canada	25898	2628	12344/13554	I-IV	67.7	Clinical symptoms	conditional logistic regression
Grieco, M. (32)	2021	cohort study	Italy	74	14	49/25	0-IV	70.6	Clinical symptoms	logistic regression
Husarić, E. (35)	2016	cohort study	Bosnia and Herzegovina	284	39	213/71	II-IV	–	Both clinical symptoms and imaging findings	logistic regression
Kim, C. H. (36)	2014	cohort study	Korea	1787	105	1159/628	0-IV	65.3	Both clinical symptoms and imaging findings	logistic regression
Morimoto, Y. (38)	2019	cohort study	Japan	417	18	259/158	I-IV	64.9	Both clinical symptoms and imaging findings	logistic regression
Nakajima, J. (50)	2010	cohort study	Japan	1004	45	566/438	0-IV	69	Clinical symptoms	logistic regression
Nakamura, Y. (39)	2021	cohort study	Japan	706	43	392/314	0-IV	70	Both clinical symptoms and imaging findings	logistic regression
Rybakov, E. G. (27)	2017	cohort study	Russia	300	39	152/148	0-IV	62.7	Both clinical symptoms and imaging findings	logistic regression
Seo, G. H. (43)	2018	cohort study	Korea	24645	3083	14003/10642	I-IV	64.1	Clinical symptoms	logistic regression
Shin, J. Y. (45)	2008	cohort study	Korea	504	41	272/232	I-IV	61.1	Both clinical symptoms and imaging findings	logistic regression
Shin, J. Y (44)	2010	cohort study	Korea	735	47	426/309	III-IV	63	Both clinical symptoms and imaging findings	logistic regression
Suwa, K. (46)	2018	cohort study	Japan	180	23	116/64	I-IV	64.9	Both clinical symptoms and imaging findings	logistic regression
Tang, L. (47)	2018	cohort study	China	3472	253	1805/1667	I-III	–	Both clinical symptoms and imaging findings	logistic regression
Namba, Y. (14)	2021	cohort study	Japan	356	48	193/163	–	73.6	Both clinical symptoms and imaging findings	logistic regression
Hu, Q. (34)	2022	cohort study	Japan	238	33	144/94	III-IV	–	Clinical symptoms	logistic regression
Matsui, R. (37)	2022	cohort study	Japan	436	94	265/171	0-IV	66.71	Both clinical symptoms and imaging findings	logistic regression
Nakamura, Y. (40)	2022	cohort study	Japan	474	31	263/211	II-IV	–	Both clinical symptoms and imaging findings	logistic regression
Ocaña, J. (26)	2022	cohort study	Spain	58	12	42/16	–	70	Both clinical symptoms and imaging findings	logistic regression
Sasaki, M. (42)	2022	cohort study	Japan	213	21	126/87	0-IV	72	Both clinical symptoms and imaging findings	logistic regression
Fujiyoshi, S. (31)	2023	cohort study	Japan	484	19	267/217	0-IV	67.8	Clinical symptoms	logistic regression
Honjo, K. (33)	2023	cohort study	Japan	1646	67	936/710	0-IV	67	Both clinical symptoms and imaging findings	logistic regression
Prassas, D. (41)	2023	cohort study	Germany	136	18	93/43	I-IV	62.76	Clinical symptoms	logistic regression
Uchida, F. (48)	2023	cohort study	Japan	1986	97	1062/924	I-IV	72.5	Clinical symptoms	logistic regression
Yanagisawa, T. (28)	2023	cohort study	Japan	134	16	90/44	0-IV	71	Clinical symptoms	logistic regression
Cai, W. T. (22)	2024	cohort study	China	1470	198	876/594	I-IV	59	–	logistic regression
Emile, S. H. (29)	2024	Case-Control Study	USA	270	36	135/135	I-IV	68.7	Both clinical symptoms and imaging findings	binary logistic regression
Yehaiya, M. (49)	2024	cohort study	China	218	42	125/93	I-IV	52.31	Both clinical symptoms and imaging findings	logistic regression
Kim, S. (25)	2025	cohort study	Korea	1202	46	639/563	I-IV	60	Both clinical symptoms and imaging findings	linear regression

### 3.3 Quality assessment

The methodological quality of the 30 included observational studies was assessed using the Newcastle-Ottawa Scale (NOS), which has a maximum score of 9 points. Studies scoring 7 points or higher are generally considered high-quality. The evaluation results showed that one study (25) scored 7 points, while the remaining 29 studies (14, 22–25, 27–50) scored between 8 and 9 points, indicating that the overall methodological quality of the included studies was high. Detailed quality assessment results are presented in **Table 2**.

**Table 2:** Risk of bias assessment of included studies.

Case control									
Study	Is the case definition adequate?	Representativeness of the cases	Determination of control group	Definition of Controls	Comparability of cases and controls based on the design or analysis	Ascertainment of exposure	Same method of ascertainment for cases and controls	Non response	Total scores
Emile, S.H. (29)	*	*	*	*	**	*	*	*	9
Cohort study									
Study	Representativeness of the exposed group	Selection of non-exposed groups	Determination of exposure factors	Identification of outcome indicators not yet to be observed at study entry	Comparability of exposed and unexposed groups considered in design and statistical analysis	design and statistical analysis	Adequacy of the study’s evaluation of the outcome	Adequacy of follow-up in exposed and unexposed groups	Total scores
Campana, J. (23)	*	*	*	0	**	*	*	*	8
Eto, K. (30)	*	*	*	*	**	*	*	*	9
Garfinkle, R. (24)	*	*	*	0	**	*	*	*	8
Grieco, M. (32)	*	*	*	0	**	*	*	*	8
Husarić, E. (35)	*	*	*	*	**	*	*	*	9
Kim, C. H. (36)	*	*	*	*	**	*	*	*	9
Morimoto, Y. (38)	*	*	*	0	**	*	*	*	8
Nakajima, J. (50)	*	*	*	*	**	*	*	*	9
Nakamura, Y. (39)	*	*	*	*	**	*	*	*	9
Rybakov, E.G. (27)	*	*	*	*	**	*	0	*	8
Seo, G.H. (43)	*	*	*	0	*	*	*	*	8
Shin, J.Y. (45)	*	*	*	*	**	*	*	*	9
Shin, J. Y 2010	*	*	*	*	**	*	*	*	9
Suwa, K. (46)	*	*	*	*	**	*	*	*	9
Tang, L. (47)	*	*	*	*	**	*	*	*	9
Namba, Y. (14)	*	*	*	*	**	*	0	*	8
Hu, Q. (34)	*	*	*	0	**	*	*	*	8
Matsui, R. (37)	*	*	*	*	**	*	*	*	9
Nakamura, Y. (40)	*	*	*	0	**	*	*	*	8
Ocaña, J. (26)	*	*	*	0	**	*	*	0	7
Sasaki, M. (42)	*	*	*	0	**	*	*	*	8
Fujiyoshi, S. (31)	*	*	*	*	**	*	*	*	9
Honjo, K. (33)	*	*	*	*	**	*	*	*	9
Prassas, D. (41)	*	*	*	*	**	*	*	*	9
Uchida, F. (48)	*	*	*	0	**	*	*	*	8
Yanagisawa, T. (28)	*	*	*	0	**	*	*	*	8
Cai, W.T. (22)	*	*	*	*	**	*	*	*	9
Yehaiya, M. (49)	*	*	*	*	**	*	*	*	9
Kim, S. (25)	*	*	*	0	**	*	*	*	8

* indicates one point awarded for the criterion. ** indicates two points awarded for the criterion. Total score is the sum of all points and reflects the methodological quality of the study (higher scores indicate higher quality).

### 3.4 Meta-analysis results

#### 3.4.1 Prevalence of postoperative ileus

Thirty studies reported the prevalence of POI in colorectal cancer patients. Analysis using a random-effects model yielded a POI prevalence of [ES = 9%, 95% CI: 8%–11%]. Due to significant heterogeneity among included studies (I^2^ = 96.80), subgroup analyses were conducted by country, TNM stage, and POI diagnostic criteria to investigate the sources of heterogeneity. Stratified by country, Chinese studies reported the highest POI prevalence (13%, 95% CI: 7%–19%), while Japan (8%, 95% CI: 6%–10%) and South Korea (7%, 95% CI: 3%–12%) reported relatively lower rates. Stratification by TNM stage revealed the lowest incidence rate (8%, 95% CI: 6%–10%) in studies including stages 0–IV, 10% (95% CI: 8%–12%) in studies including stages I–IV, and 10% (95% CI: 3%–17%) in studies including stages II–IV or III–IV.

Subgroup analysis based on POI diagnostic criteria revealed that overall prevalence estimates were similar between studies relying solely on clinical symptoms (9%, 95% CI: 7%–11%) and those combining clinical symptoms with imaging findings (9%, 95% CI: 8%–11%), with overlapping confidence intervals. To further investigate the sources of heterogeneity, we conducted a meta-regression analysis using country, TNM staging distribution, and POI diagnostic criteria as covariates. As shown in **Table 3**, none of these covariates were significantly associated with the combined prevalence of POI (P > 0.05). **Table 4** presents detailed results of subgroup analyses. However, no sources of heterogeneity were identified in these analyses. Sensitivity analyses conducted by sequentially excluding individual studies showed no significant changes in effect size pooling, indicating stable results. Publication bias was assessed using Egger’s test and funnel plots. The Egger test (P = 0.35) indicated no significant publication bias. Although the funnel plot showed asymmetry, trim-and-trim analysis suggested no need to include missing studies, and the adjusted effect size remained unchanged. Thus, the overall results were minimally affected by publication bias, and the conclusions are robust. The corresponding forest plot, sensitivity analysis, Egger’s test, funnel plot, and trim-and-fill results are presented in **Supplementary Material 2** (**Supplementary Figures S1**-**S5**).

**Table 3:** Meta-regression analysis of factors associated with POI prevalence.

Variables	Coefficient	95%CI	Std. Err	P value
Argentina	0.97	(0.88,1.08)	0.05	0.55
Bosnia and Herzegovina	1.04	(0.94,1.16)	0.06	0.43
Canada	1.00	(0.90,1.11)	0.05	0.92
Germany	1.04	(0.92,1.17)	0.06	0.53
Italy	1.10	(0.96,1.26)	0.07	0.16
Japan	0.98	(0.94,1.01)	0.02	0.18
Russia	1.04	(0.93,1.15)	0.05	0.52
Spain	1.12	(0.97,1.30)	0.08	0.13
America	1.04	(0.93,1.16)	0.06	0.48
China	1.04	(0.97,1.10)	0.03	0.25
Korea	0.97	(0.93,1.02)	0.02	0.24
0-IV	0.99	(0.95,1.02)	0.02	0.45
I-III	0.98	(0.88,1.08)	0.05	0.62
I-IV	1.00	(0.97,1.04)	0.02	0.84
III-IV	1.00	(0.93,1.08)	0.04	0.97
II-IV	1.00	(0.93,1.08)	0.04	0.94
Clinical symptoms	1.00	(0.96,1.03)	0.02	0.84
Both symptoms and imaging findings	1.00	(0.96,1.03)	0.02	0.92

**Table 4:** Subgroup analysis for postoperative ileus rates.

Outcome	Group	Subgroup	No. of study	Heterogeneity (%)	ES 95%CI
Incidence of postoperative ileus	Country	Japan	14	90.6	8% (6%-10%)
		Korea	5	98.8	7% (3%-12%)
		China	3	96.3	13% (7%-19%)
	TNM	I-IV	12	97.6	10% (8%-12%)
		0-IV	11	91.5	8% (6%-10%)
		II-IV	2	89.4	10% (3%-17%)
		III-IV	2	89.6	10% (3%-17%)

#### 3.4.2 Male

**Figure 2:**
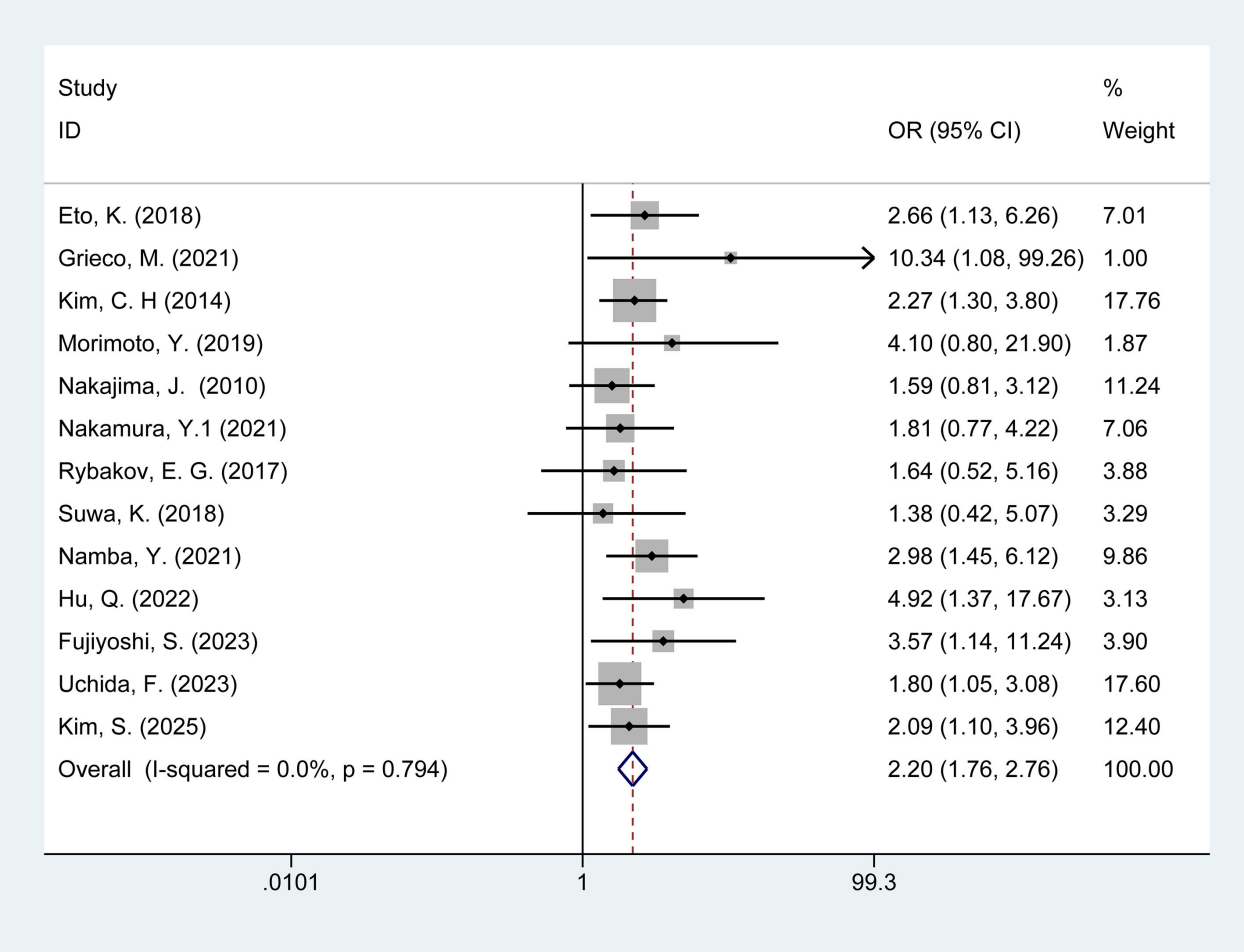
Forest plot of the meta-analysis of male.

Thirteen studies examined the association between male and POI in colorectal cancer patients. No heterogeneity was observed among these studies (I^2^ = 0%, p = 0.79). Therefore, data were pooled using a fixed-effect model. Meta-analysis results (**Figure 2**) indicate that male gender is associated with a risk of POI in colorectal cancer patients [OR = 2.20, 95% CI: 1.76–2.76].

#### 3.4.3 Operating time>3 hours

**Figure 3:**
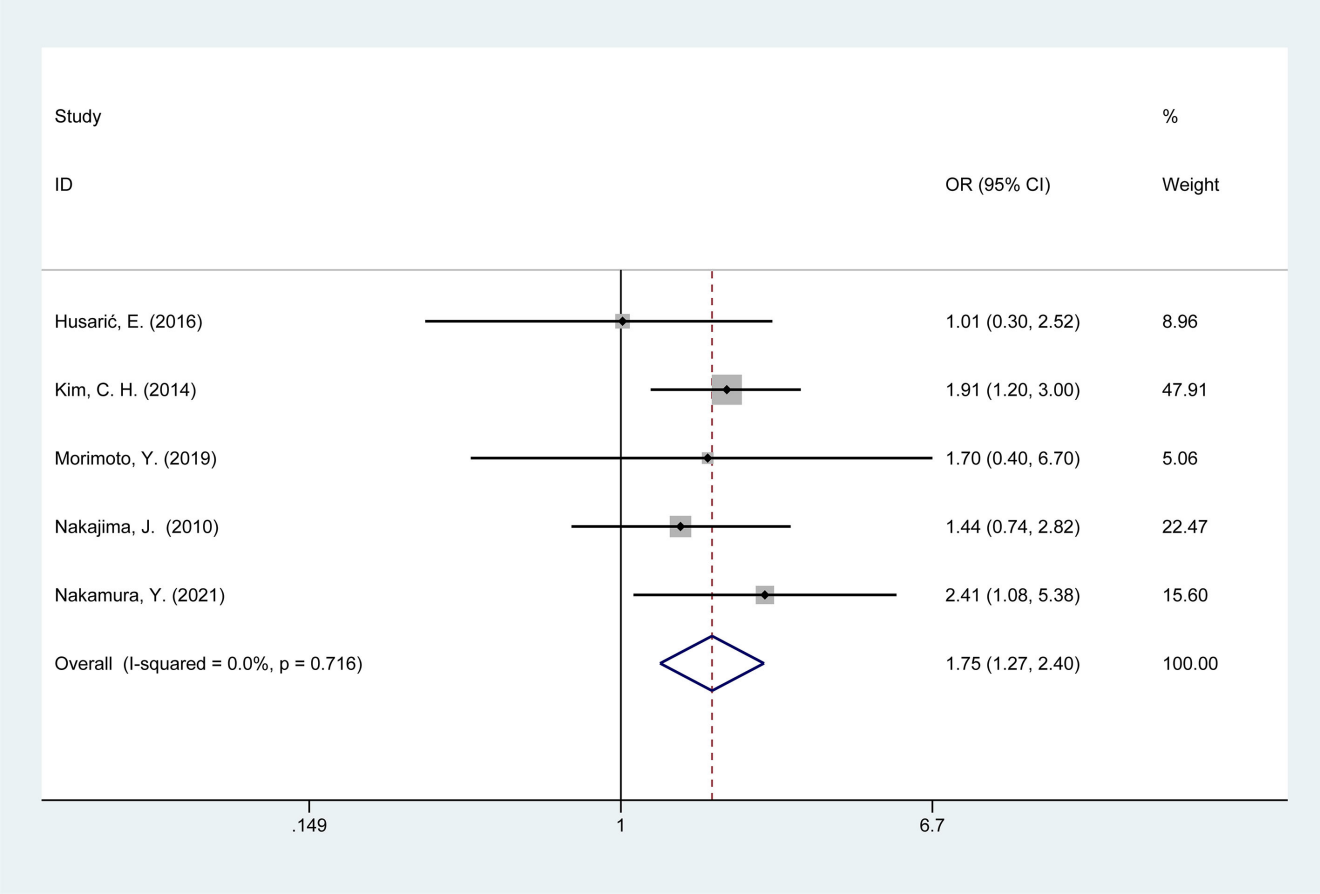
Forest plot of the meta-analysis of operative time > 3 hours.

Five studies examined the association between operative time >3 hours and POI in colorectal cancer patients. No heterogeneity was found among these studies (I^2^ = 0%, p = 0.72); therefore, the data were pooled using a fixed-effects model. Meta-analysis results (**Figure 3**) indicate that a surgical duration exceeding 3 hours is associated with a risk of POI in colorectal cancer patients [OR = 1.75, 95% CI: 1.27–2.40].

#### 3.4.4 Open surgery

**Figure 4:**
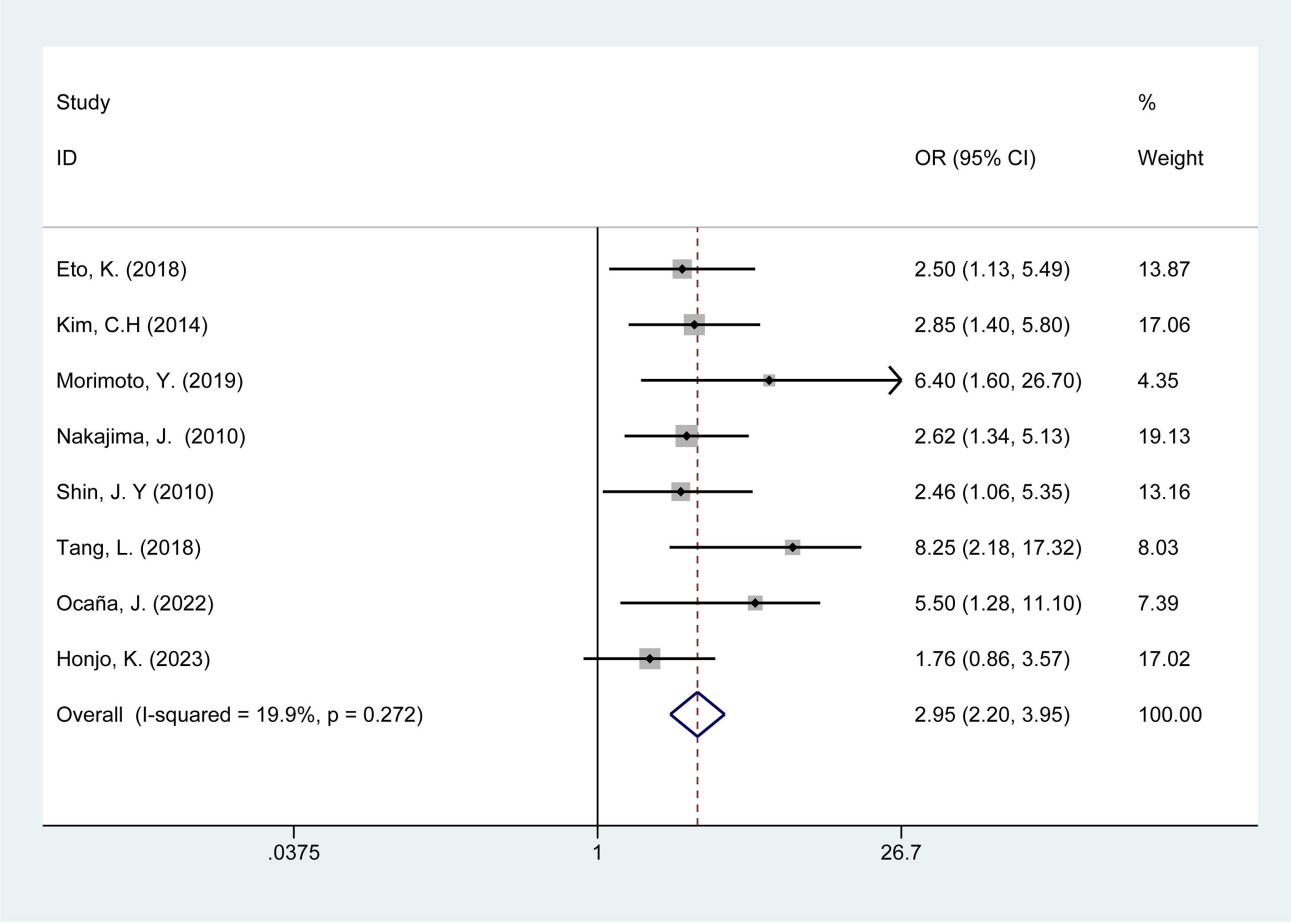
Forest plot of the meta-analysis of open surgery.

Eight studies compared the effects of POI in patients with colorectal cancer undergoing open surgery. These studies exhibited low heterogeneity (I^2^ = 19.9%, p = 0.27), allowing for data pooling using a fixed-effect model. Meta-analysis results (**Figure 4**) indicate that open surgery is associated with a risk of POI in colorectal cancer patients [OR = 2.95, 95% CI: 2.20–3.95]. Sensitivity analysis was performed using a stepwise exclusion method, and the results remained stable. Analysis details are provided in **Supplementary Material 2**, **Supplementary Figure S6**.

#### 3.4.5 Ileostomy

**Figure 5:**
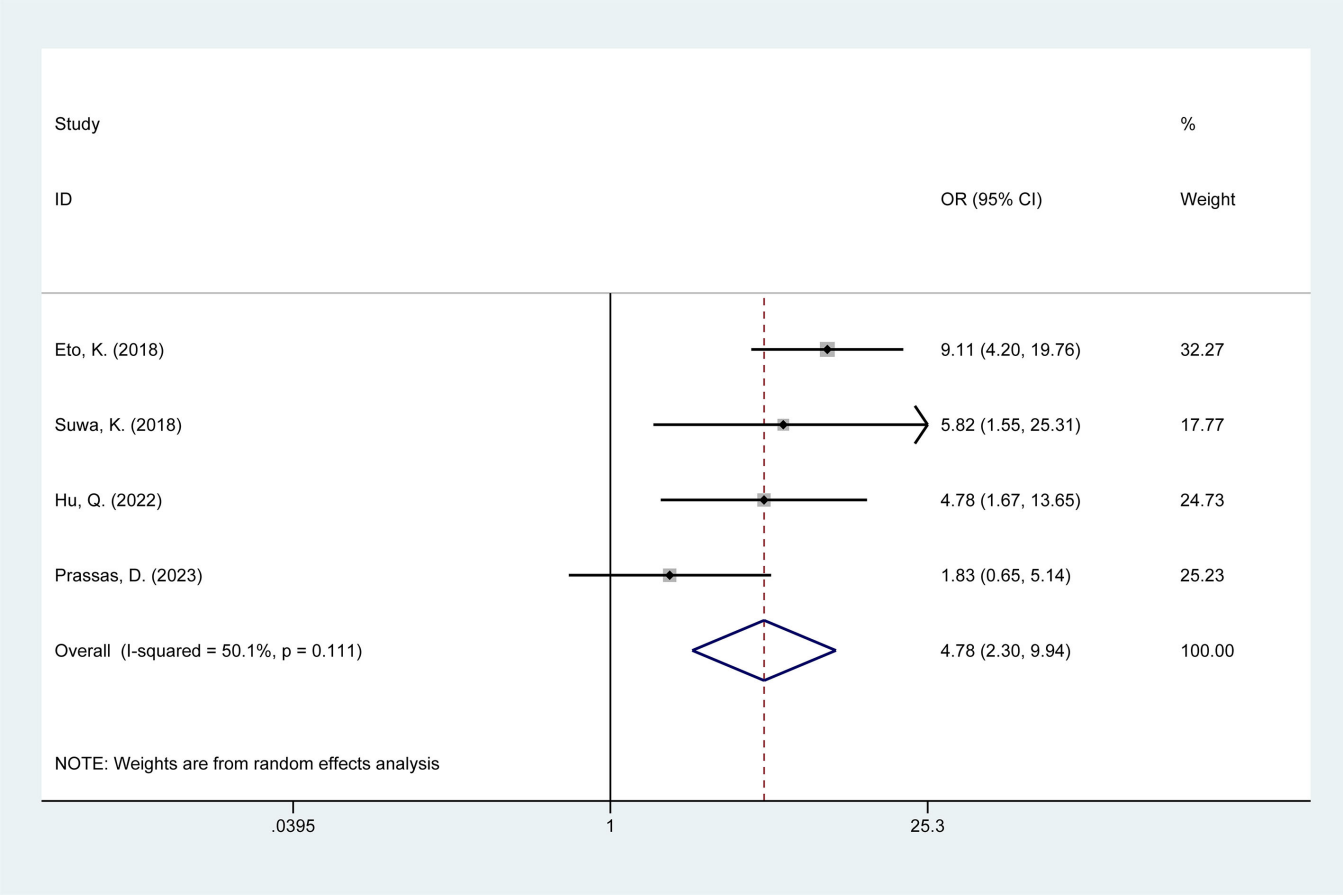
Forest plot of the meta-analysis of ileostomy.

Four studies examined the impact of ileostomy on POI in colorectal cancer patients. Heterogeneity existed among these studies (I^2^ = 50.10%, p = 0.11); thus, data were pooled using a random-effects model. Meta-analysis results (**Figure 5**) indicate that ileostomy is associated with a risk of POI following colorectal cancer surgery [OR = 4.78, 95% CI: 2.30–9.94]. Due to substantial heterogeneity, sensitivity analysis was performed using a stepwise exclusion method, revealing stable results. Analysis details are presented in **Supplementary Material 2**, **Supplementary Figure S7**.

#### 3.4.6 Previous abdominal surgery

**Figure 6:**
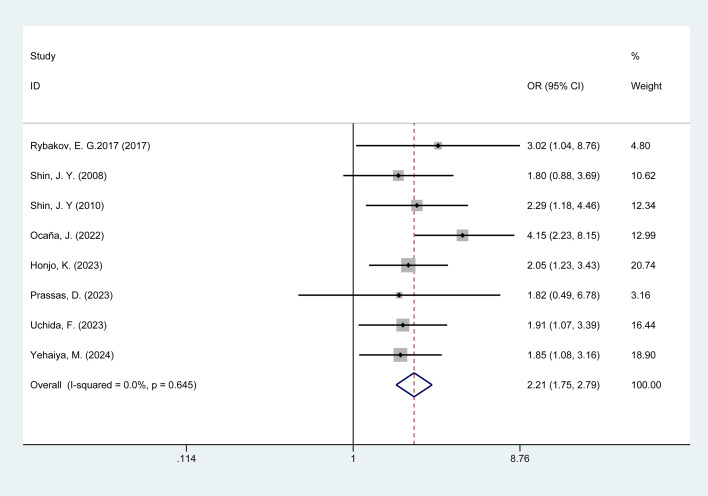
Forest plot of the meta-analysis of previous abdominal surgery.

Eight studies examined the association between previous abdominal surgery and POI in colorectal cancer patients. No heterogeneity was observed among these studies (I^2^ = 0%, p = 0.65); thus, data were pooled using a fixed-effects model. Meta-analysis results (**Figure 6**) indicate that Previous abdominal surgery is associated with a risk of POI in colorectal cancer patients [OR = 2.21, 95% CI: 1.75–2.79].

#### 3.4.7 Age≥65 years

**Figure 7:**
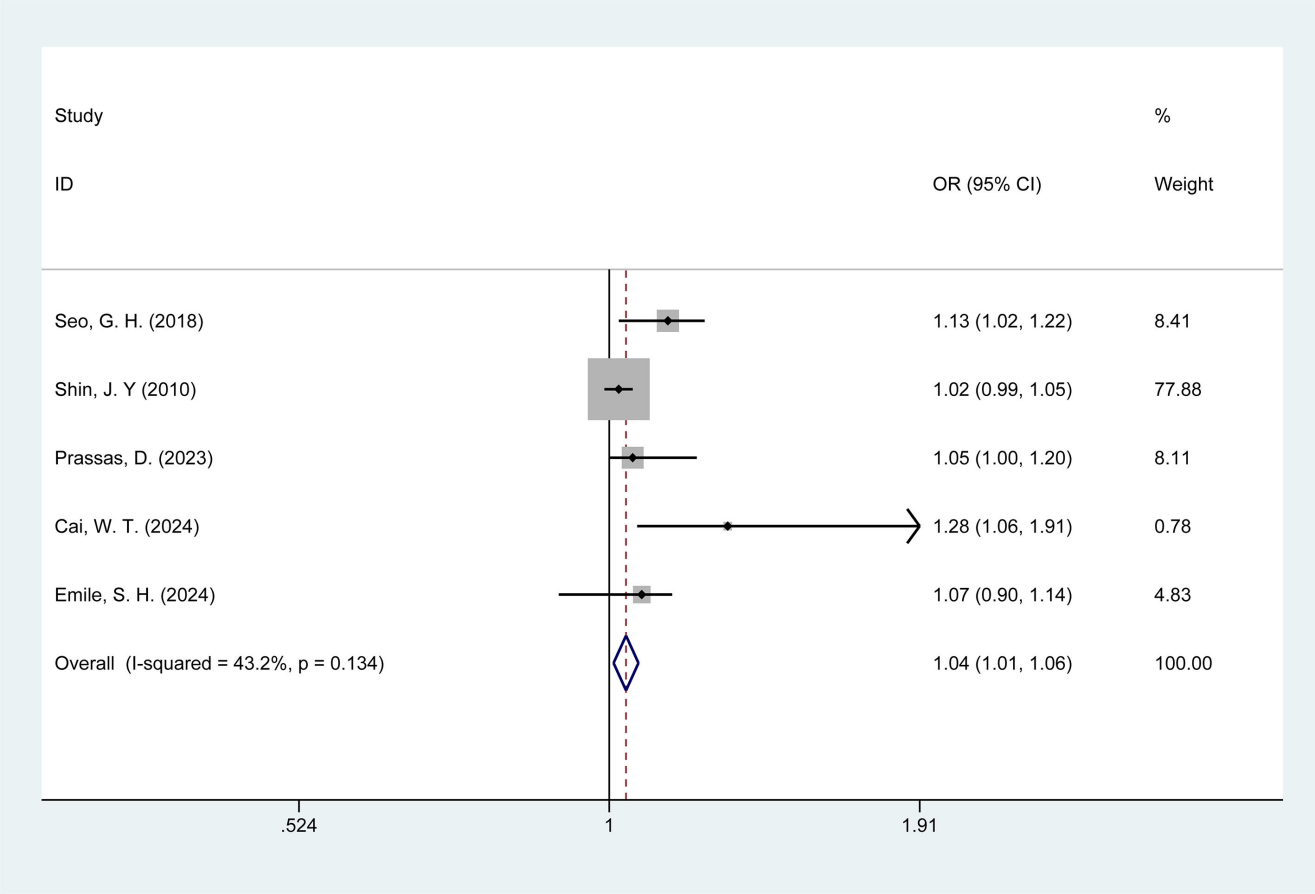
Forest plot of the meta-analysis of age≥65.

Five studies examined the association between age≥ 65 years and POI in colorectal cancer patients. No significant heterogeneity was observed among these studies (I^2^ = 43.20%, p = 0.13); therefore, the data were pooled using a fixed-effects model. Meta-analysis results (**Figure 7**) indicate that age ≥ 65 years may be associated with a risk of POI in colorectal cancer patients [OR = 1.04, 95% CI: 1.01–1.06].

#### 3.4.8 Publication bias results

Egger’s test indicated potential publication bias for open surgery (p = 0.02), with funnel plots showing asymmetry. However, the trim-and-fill analysis demonstrated no need to include missing studies, and the adjusted effect size remained unchanged, suggesting a minimal overall impact of publication bias and robust conclusions. No significant bias was detected for other factors: male gender (p=0.06), age≥65 years (p=0.06), operative time >3 hours (p=0.54), ileostomy (p=0.56), or previous abdominal surgery (p=0.67). All funnel plots, Egger’s tests, and trim-and-trim results are presented in **Supplementary Material 2** (**Supplementary Figure S8**-**S20**).

## 4 Discussion

This systematic review and meta-analysis evaluated 30 studies involving 73,433 patients with colorectal cancer, among whom 7,463 experienced POI, yielding an overall incidence rate of [ES = 9%, 95% CI: 8%–11%]. Meta-analysis results suggest that male gender, operative time >3 hours, open surgery, ileostomy, previous abdominal surgery, and age≥65 years are associated with POI in colorectal cancer patients. However, methodological variability across the included studies should be taken into account when interpreting these findings.

### 4.1 Prevalence of POI

This meta-analysis indicates that the prevalence of POI among colorectal cancer patients is 9%, representing a significant disease burden. The study found that the reported prevalence of POI varied depending on the stage range of included patients. Studies primarily enrolling advanced-stage patients (stage II-IV or III-IV) reported higher incidence rates than those including all stages (0-IV). Although the limited number of studies reporting data on advanced-stage patients may contribute to imprecise results, this trend suggests that populations with a higher proportion of mid-to-late-stage patients may exhibit a greater observed risk of POI. This may relate to the greater complexity of surgery, increased trauma, and stronger inflammatory responses associated with advanced-stage tumors.

When analyzing inter-country variations, Chinese studies reported the highest prevalence rate of 13%, exceeding those from Japan and South Korea. However, given the limited number of Chinese studies included, this finding warrants cautious interpretation. Such regional differences may stem from variations in the baseline characteristics of the study populations, potentially due to an uneven distribution of tumor stages among enrolled patients, rather than necessarily reflecting geographical factors. Additionally, differences in surgical practice patterns across countries, particularly variations in the proportion of minimally invasive procedures, may be a significant contributor to this heterogeneity. Minimally invasive surgery is associated with reduced surgical trauma and accelerated postoperative gastrointestinal recovery, which may lower the risk of POI. Unfortunately, detailed information regarding surgical techniques was not consistently reported across the included studies, precluding further subgroup analyses. Consequently, the observed inter-country differences are more likely to reflect variations in patient and treatment characteristics rather than actual geographic effects.

Due to significant variations in POI diagnostic criteria across different studies, we conducted subgroup analyses to investigate their impact on prevalence estimates. Results showed similar overall prevalence of POI between subgroups based on different diagnostic approaches (clinical symptoms alone vs. clinical symptoms combined with imaging findings), suggesting that differences in diagnostic criteria themselves may not substantially affect prevalence estimates at the study level.

To further investigate sources of heterogeneity, we performed meta-regression analysis incorporating covariates such as country, TNM staging distribution, and POI diagnostic criteria. The results did not identify any of these factors as significantly associated with heterogeneity. Collectively, the significant heterogeneity observed in this study is likely not attributable to a single dominant factor, but rather the result of multiple factors acting in concert. These factors include, but are not limited to, variations in surgical techniques, differences in perioperative management pathways, patient comorbidities, and inconsistencies in clinical practice across different healthcare institutions. These factors are often inadequately measured or reported inconsistently in the original studies.

### 4.2 Association between male and POI

This study found an association between male sex and POI. This conclusion aligns with multiple studies. A cohort study involving 5,369 patients indicated that the probability of POI occurrence in males was nearly twice that of females (51). Additionally, research by Koch KE et al. also revealed a significantly increased risk of POI in males (OR = 1.97) (52). Possible reasons include the following: The relatively narrow male pelvic structure limits operative space during low rectal resection or pelvic surgery, making the procedure more complex (53). This often leads to prolonged surgery, increased risk of excessive bowel traction and tissue injury, thereby exacerbating postoperative inflammatory responses and raising the risk of POI (53–55). Second, the male pelvic anatomy predisposes the inferior hypogastric nerves and pelvic plexus to injury during surgery (56). These autonomic nerves play a crucial role in maintaining intestinal motility; their damage may delay intestinal function recovery, further increasing the likelihood of POI (57).

Additionally, male patients frequently present with high-risk factors such as obstructive pulmonary disease and smoking history. The synergistic effects of these factors may also elevate the risk of POI (31, 58). Notably, the higher proportion of visceral fat in males also increases surgical difficulty and inflammatory response levels (38). Given that male patients are more prone to postoperative POI following colorectal cancer surgery, it is recommended to conduct a comprehensive preoperative risk assessment, evaluating factors such as chronic obstructive pulmonary disease (COPD), smoking history, and obesity. Enhanced intraoperative neuroprotection and perioperative management should be implemented, in conjunction with the active application of an Enhanced Recovery After Surgery (ERAS) protocol, to reduce the risk of POI.

### 4.3 Association between operating time (>3 hours) and POI

This study found that operating times exceeding 3 hours were associated with POI. This finding aligns with the results of large-scale observational studies by Chapuis et al (59). This association may stem from several factors: First, prolonged surgery typically indicates a more extensive surgical scope or greater procedural complexity, potentially leading to more significant tissue trauma and inflammatory cytokine release (60, 61). This, in turn, may suppress intestinal neural function and delay the recovery of peristalsis (18). Simultaneously, prolonged surgery inevitably involves increased anesthetic drug dosage and duration, leading to heightened release of inhibitory neurotransmitters like acetylcholine. This further suppresses intestinal smooth muscle function, prolongs paralytic ileus, and elevates the risk of POI (62). Notably, surgical duration is not an isolated factor; it often reflects a combination of patient complexity, tumor anatomical challenges, and surgeon proficiency. Therefore, it is recommended to reasonably control surgical time while ensuring quality by enhancing the surgical team’s expertise, prioritizing minimally invasive techniques, and optimizing perioperative management to minimize the risk of postoperative ileus.

### 4.4 Association between open surgery and POI

This study found that open surgery was associated with POI in colorectal cancer patients, a finding that is consistent with multiple previous cohort studies and systematic reviews (55, 63, 64). This may be attributed to the greater physical trauma associated with open surgery: prolonged exposure of intestinal segments and increased intraoperative blood loss exacerbate the body’s stress response and activate the immune system. This promotes the release of multiple inflammatory mediators, leading to intestinal wall congestion and edema, fibrin exudation, and impaired intestinal motility, thereby increasing the risk of intra-abdominal infection and adhesion formation (65, 66). Stommel et al.’s study also demonstrated a lower incidence of abdominal wall adhesions with laparoscopic surgery, further supporting the findings of this research (67). In contrast, laparoscopic surgery, due to its minimally invasive nature, reduces tissue damage and local inflammatory responses, shortens the duration of postoperative ileus, and promotes faster patient recovery (68). Therefore, when feasible, minimally invasive surgical approaches should be considered as part of strategies to reduce the risk of POI.

### 4.5 Association between ileostomy and POI

This study found that ileostomy was associated with POI in colorectal cancer patients, consistent with findings from multiple previous studies. Millan et al.’s retrospective database analysis explicitly identified ileostomy as an independent risk factor for POI (69). Similarly, Reichert et al.’s research demonstrated that patients undergoing ileostomy had a significantly elevated risk of POI—up to five times higher—compared to those without stoma creation (70). Furthermore, a meta-analysis of randomized controlled trials revealed that early stoma removal significantly reduces the incidence of POI, indirectly supporting the positive correlation between stoma retention duration and POI risk (71). Potential mechanisms include: First, stoma creation itself involves additional bowel manipulation and traction, leading to more pronounced peritoneal and mesenteric trauma. This exacerbates local inflammatory responses and delays the recovery of intestinal motility (72). Second, stomas are often associated with significant fluid loss and electrolyte imbalances, which may further exacerbate intestinal dysfunction. In clinical practice, these findings suggest the need for careful assessment of stoma necessity (73, 74). For patients requiring an ileostomy, intraoperative measures should minimize bowel manipulation and traction, optimize fluid management, and enhance postoperative electrolyte and nutritional support. Additionally, exploring earlier stoma reversal in suitable patients is recommended to potentially reduce the risk of long-term postoperative ileus.

### 4.6 Association between previous abdominal surgery and POI

This study found that previous abdominal surgery was associated with POI in colorectal cancer patients. This finding aligns with the results of a meta-analysis by Quiroga-Centeno et al. (75). Potential contributing factors include: First, trauma, inflammatory responses, and retained foreign bodies (such as sutures) from previous abdominal surgery can induce intestinal adhesions (76). Even if the current colorectal cancer surgery is completed successfully, pre-existing adhesions or altered local anatomy may still increase surgical difficulty and duration (77). Furthermore, extensive tissue dissection and prolonged exposure and traction of the bowel can exacerbate damage to the intestinal serosal surface and inflammatory response, significantly delaying the recovery of intestinal motility and increasing the risk of POI or worsening its severity (78, 79). Therefore, for colorectal cancer patients with previous abdominal surgery, minimally invasive surgery should be prioritized. Intraoperatively, efforts should focus on minimizing bowel injury and unnecessary traction. Postoperatively, an ERAS pathway should be implemented to actively promote bowel function recovery, thereby reducing the risk of POI.

### 4.7 Association between age≥65 years and POI

This meta-analysis indicates an association between age ≥65 years and POI, although the association strength is low (OR = 1.04), suggesting limited clinical significance. This finding suggests that age itself may not be the primary determinant of POI risk, but rather likely reflects a composite of multiple underlying risk characteristics in elderly patients. These include diminished physiological reserves and nutritional status (anemia, hypoalbuminemia) (80, 81), increased burden of chronic comorbidities (cardiovascular disease, chronic obstructive pulmonary disease, metabolic disorders) (82, 83), and abnormal inflammatory responses (84). These factors not only elevate perioperative complication risks but may also increase analgesic requirements (opioids), further delaying bowel recovery (85, 86).

Previous studies have similarly indicated a moderate association between age and POI (59, 81), with the underlying mechanism likely stemming from increased comorbidities and diminished surgical stress tolerance associated with ageing, rather than age itself directly causing POI. Therefore, while perioperative management for elderly patients requires greater caution, clinical interventions should prioritize modifiable risk factors that can be addressed. These include optimizing nutritional status, minimizing surgical trauma, enhancing pain management, and implementing an accelerated recovery from surgery (ERAS) protocol early. Compared to focusing solely on age, this comprehensive management strategy offers greater clinical value in reducing the risk of POI.

### 4.8 Strengths and limitations

The study strictly adhered to the inclusion and exclusion criteria, incorporating only studies that employed multivariate regression analysis, thereby mitigating the influence of confounding factors to some extent. Furthermore, the included studies were of moderate to high quality, ensuring the reliability of the results. Additionally, sensitivity analyses and publication bias assessments were conducted to validate the findings, enhancing their robustness.

Despite these efforts, the present study has several limitations. First, although only studies employing multivariate regression analysis were included to mitigate confounding effects, inconsistencies in reporting and missing data were noted for certain critical perioperative variables across the included studies. These variables included adherence to ERAS protocols, opioid exposure, BMI, burden of comorbidities, and tumor location. Consequently, reliable integration of these variables into the current analysis was not feasible. Second, differences in POI diagnostic criteria and covariate adjustment strategies across studies may have introduced residual heterogeneity and potential confounding bias into the results.

Furthermore, the number of studies examining certain specific risk factors remains limited, and the evidence for these remains insufficient. Future research should prioritize rigorously designed, large-scale prospective or multicenter studies that employ standardized POI definitions and systematically collect and report key perioperative variables. This will help further clarify the independent impact of individual factors on POI, enable more precise risk stratification, and provide higher-level evidence-based support for developing targeted interventions to reduce POI risk.

## 5 Conclusion

This meta-analysis examined the prevalence and risk factors associated with postoperative ileus (POI) in colorectal cancer patients. The results indicate that the overall prevalence of POI following colorectal cancer surgery is 9% (95% CI: 8%–11%), suggesting that POI remains a common and clinically significant complication after colorectal cancer surgery. The meta-analysis further suggests that male, operative time >3 hours, open surgery, ileostomy, previous abdominal surgery, and age≥65 years may be potential risk factors for POI in colorectal cancer patients. Therefore, identifying patients with these characteristics during the perioperative period may help inform risk stratification and guide the implementation of targeted preventive strategies to potentially reduce the occurrence of POI.

## Data Availability

The original contributions presented in the study are included in the article/**Supplementary Material**. Further inquiries can be directed to the corresponding author.
