# Supplementary material for: Prevalence and risk factors for postoperative ileus in colorectal cancer patients: a systematic review and meta-analysis
Source: Front Oncol. 2026 Jan 16;15:1742152. doi: 10.3389/fonc.2025.1742152 (PMC12855047; doi:10.3389/fonc.2025.1742152)
Supplement: Supplementary file 1 [file DataSheet1.docx]

PubMed

| Search  number | Query | Results |
| --- | --- | --- |
| 1 | "Colorectal Neoplasms"[Mesh] | 258,093 |
| 2 | (((((((((((((((Colorectal Neoplasms[Title/Abstract]) OR (Colorectal Neoplasm[Title/Abstract])) OR (Neoplasm, Colorectal[Title/Abstract])) OR (Colorectal Tumors[Title/Abstract])) OR (Colorectal Tumor[Title/Abstract])) OR (Tumor, Colorectal[Title/Abstract])) OR (Tumors, Colorectal[Title/Abstract])) OR (Neoplasms, Colorectal[Title/Abstract])) OR (Colorectal Cancer[Title/Abstract])) OR (Cancer, Colorectal[Title/Abstract])) OR (Cancers, Colorectal[Title/Abstract])) OR (Colorectal Cancers[Title/Abstract])) OR (Colorectal Carcinoma[Title/Abstract])) OR (Carcinoma, Colorectal[Title/Abstract])) OR (Carcinomas, Colorectal[Title/Abstract])) OR (Colorectal Carcinomas[Title/Abstract]) | 174,458 |
| 3 | #1 OR #2 | 311,754 |
| 4 | "Intestinal Obstruction"[Mesh] | 52,034 |
| 5 | (((Intestinal Obstruction[Title/Abstract]) OR (Intestinal Obstructions[Title/Abstract])) OR (Obstruction, Intestinal[Title/Abstract])) OR (Ileus[Title/Abstract]) | 31,466 |
| 6 | #4 OR #5 | 66,542 |
| 7 | "Risk Factors"[Mesh] | 1,048,508 |
| 8 | ((((((((((((((((((Risk Factors[Title/Abstract]) OR (Factor, Risk[Title/Abstract])) OR (Risk Factor[Title/Abstract])) OR (Population at Risk[Title/Abstract])) OR (Populations at Risk[Title/Abstract])) OR (Risk Scores[Title/Abstract])) OR (Risk Score[Title/Abstract])) OR (Score, Risk[Title/Abstract])) OR (Risk Factor Scores[Title/Abstract])) OR (Risk Factor Score[Title/Abstract])) OR (Score, Risk Factor[Title/Abstract])) OR (Health Correlates[Title/Abstract])) OR (Correlates, Health[Title/Abstract])) OR (Social Risk Factors[Title/Abstract])) OR (Factor, Social Risk[Title/Abstract])) OR (Factors, Social Risk[Title/Abstract])) OR (Risk Factor, Social[Title/Abstract])) OR (Risk Factors, Social[Title/Abstract])) OR (Social Risk Factor[Title/Abstract]) | 942,258 |
| 9 | #7 OR #8 | 1,576,590 |
| 10 | #3 AND #6 AND #9 | 419 |

Embase

| No. | Query | Results |
| --- | --- | --- |
| #1 | 'colorectal tumor'/exp | 542037 |
| #2 | 'colorectal neoplasms':ab,ti OR 'colorectal neoplasm':ab,ti OR 'neoplasm, colorectal':ab,ti OR 'colorectal tumors':ab,ti OR 'colorectal tumor':ab,ti OR 'tumor, colorectal':ab,ti OR 'tumors, colorectal':ab,ti OR 'neoplasms, colorectal':ab,ti OR 'colorectal cancer':ab,ti OR 'cancer, colorectal':ab,ti OR 'cancers, colorectal':ab,ti OR 'colorectal cancers':ab,ti OR 'colorectal carcinoma':ab,ti OR 'carcinoma, colorectal':ab,ti OR 'carcinomas, colorectal':ab,ti OR 'colorectal carcinomas':ab,ti | 258232 |
| #3 | #1 OR #2 | 561812 |
| #4 | 'intestine obstruction'/exp | 130205 |
| #5 | 'intestinal obstruction':ab,ti OR 'intestinal obstructions':ab,ti OR 'obstruction, intestinal':ab,ti OR 'ileus':ab,ti | 40536 |
| #6 | #4 OR #5 | 137683 |
| #7 | 'risk factor'/exp | 1596463 |
| #8 | 'risk factors':ab,ti OR 'factor, risk':ab,ti OR 'risk factor':ab,ti OR 'population at risk':ab,ti OR 'populations at risk':ab,ti OR 'risk scores':ab,ti OR 'risk score':ab,ti OR 'score, risk':ab,ti OR 'risk factor scores':ab,ti OR 'risk factor score':ab,ti OR 'score, risk factor':ab,ti OR 'health correlates':ab,ti OR 'correlates, health':ab,ti OR 'social risk factors':ab,ti OR 'factor, social risk':ab,ti OR 'factors, social risk':ab,ti OR 'risk factor, social':ab,ti OR 'risk factors, social':ab,ti OR 'social risk factor':ab,ti | 1380469 |
| #9 | #7 OR #8 | 2093948 |
| #10 | #3 AND #6 AND #9 | 1286 |

Cochrane library

| ID | Search | Hits |
| --- | --- | --- |
| #1 | MeSH descriptor: [Colorectal Neoplasms] explode all trees | 13177 |
| #2 | (Colorectal Neoplasms):ab,ti,kw OR (Colorectal Neoplasm):ab,ti,kw OR (Neoplasm, Colorectal):ab,ti,kw OR (Colorectal Tumors):ab,ti,kw OR (Colorectal Tumor):ab,ti,kw OR (Tumor, Colorectal):ab,ti,kw OR (Tumors, Colorectal):ab,ti,kw OR (Neoplasms, Colorectal):ab,ti,kw OR (Colorectal Cancer):ab,ti,kw OR (Cancer, Colorectal):ab,ti,kw OR (Cancers, Colorectal):ab,ti,kw OR (Colorectal Cancers):ab,ti,kw OR (Colorectal Carcinoma):ab,ti,kw OR (Carcinoma, Colorectal):ab,ti,kw OR (Carcinomas, Colorectal):ab,ti,kw OR (Colorectal Carcinomas):ab,ti,kw | 21986 |
| #3 | #1 OR #2 | 25505 |
| #4 | MeSH descriptor: [Intestinal Obstruction] explode all trees | 973 |
| #5 | (Intestinal Obstruction):ab,ti,kw OR (Intestinal Obstructions):ab,ti,kw OR (Obstruction, Intestinal):ab,ti,kw OR (Ileus):ab,ti,kw | 4071 |
| #6 | #4 OR #5 | 4168 |
| #7 | MeSH descriptor: [Risk Factors] explode all trees | 38053 |
| #8 | (Risk Factors):ab,ti,kw OR (Factor, Risk):ab,ti,kw OR (Risk Factor):ab,ti,kw OR (Population at Risk):ab,ti,kw OR (Populations at Risk):ab,ti,kw OR (Risk Scores):ab,ti,kw OR (Risk Score):ab,ti,kw OR (Score, Risk):ab,ti,kw OR (Risk Factor Scores):ab,ti,kw OR (Risk Factor Score):ab,ti,kw OR (Score, Risk Factor):ab,ti,kw OR (Health Correlates):ab,ti,kw OR (Correlates, Health):ab,ti,kw OR (Social Risk Factors):ab,ti,kw OR (Factor, Social Risk):ab,ti,kw OR (Factors, Social Risk):ab,ti,kw OR (Risk Factor, Social):ab,ti,kw OR (Risk Factors, Social):ab,ti,kw OR (Social Risk Factor):ab,ti,kw | 193273 |
| #9 | #7 OR #8 | 193273 |
| #10 | #3 AND #6 AND #9 | 64 |

Web of science

| ID | Search | Hits |
| --- | --- | --- |
| #1 | TS=(Colorectal Neoplasms) OR TS=(Colorectal Neoplasm) OR TS=(Neoplasm, Colorectal) OR TS=(Colorectal Tumors) OR TS=(Colorectal Tumor) OR TS=(Tumor, Colorectal) OR TS=(Tumors, Colorectal) OR TS=(Neoplasms, Colorectal) OR TS=(Colorectal Cancer) OR TS=(Cancer, Colorectal) OR TS=(Cancers, Colorectal) OR TS=(Colorectal Cancers) OR TS=(Colorectal Carcinoma) OR TS=(Carcinoma, Colorectal) OR TS=(Carcinomas, Colorectal) OR TS=(Colorectal Carcinomas) | 275185 |
| #2 | TS=(Intestinal Obstruction) OR TS=(Intestinal Obstructions) OR TS=(Obstruction, Intestinal) OR TS=(Ileus) | 19339 |
| #3 | TS=(Risk Factors) OR TS=(Factor, Risk) OR TS=(Risk Factor) OR TS=(Population at Risk) OR TS=(Populations at Risk) OR TS=(Risk Scores) OR TS=(Risk Score) OR TS=(Score, Risk) OR TS=(Risk Factor Scores) OR TS=(Risk Factor Score) OR TS=(Score, Risk Factor) OR TS=(Health Correlates) OR TS=(Correlates, Health) OR TS=(Social Risk Factors) OR TS=(Factor, Social Risk) OR TS=(Factors, Social Risk) OR TS=(Risk Factor, Social) OR TS=(Risk Factors, Social) OR TS=(Social Risk Factor) | 2142315 |
| #4 | #1 AND #2 AND #3 | 336 |
