# Supplementary material for: Prevalence and risk factors for postoperative ileus in colorectal cancer patients: a systematic review and meta-analysis
Source: Front Oncol. 2026 Jan 16;15:1742152. doi: 10.3389/fonc.2025.1742152 (PMC12855047; doi:10.3389/fonc.2025.1742152)
Supplement: Supplementary file 2 [file DataSheet2.docx]

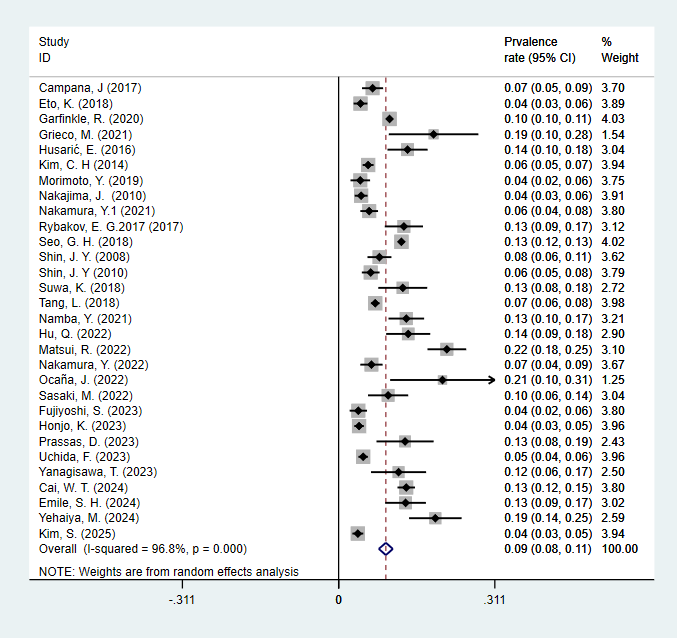


Figure S1. Forest plot of postoperative ileus incidence in colorectal cancer patients


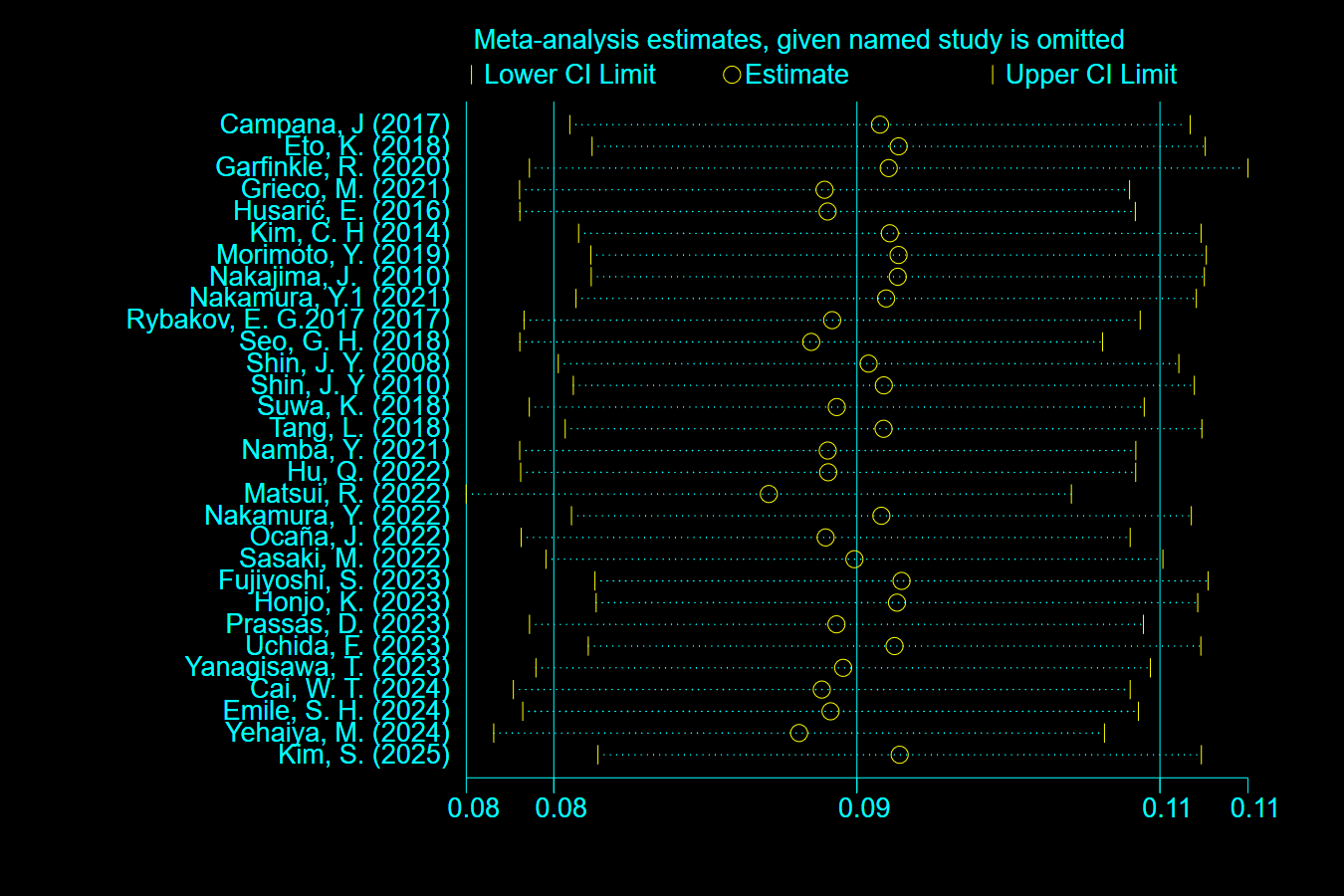


Figure S2. Sensitivity analysis of the postoperative ileus incidence rate


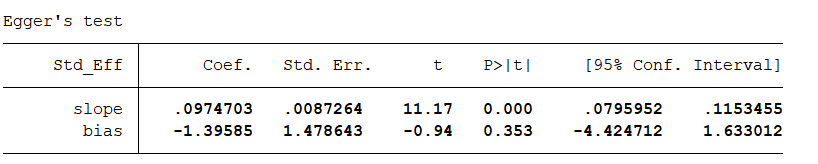


Figure S3. Egger's test for the incidence of postoperative ileus


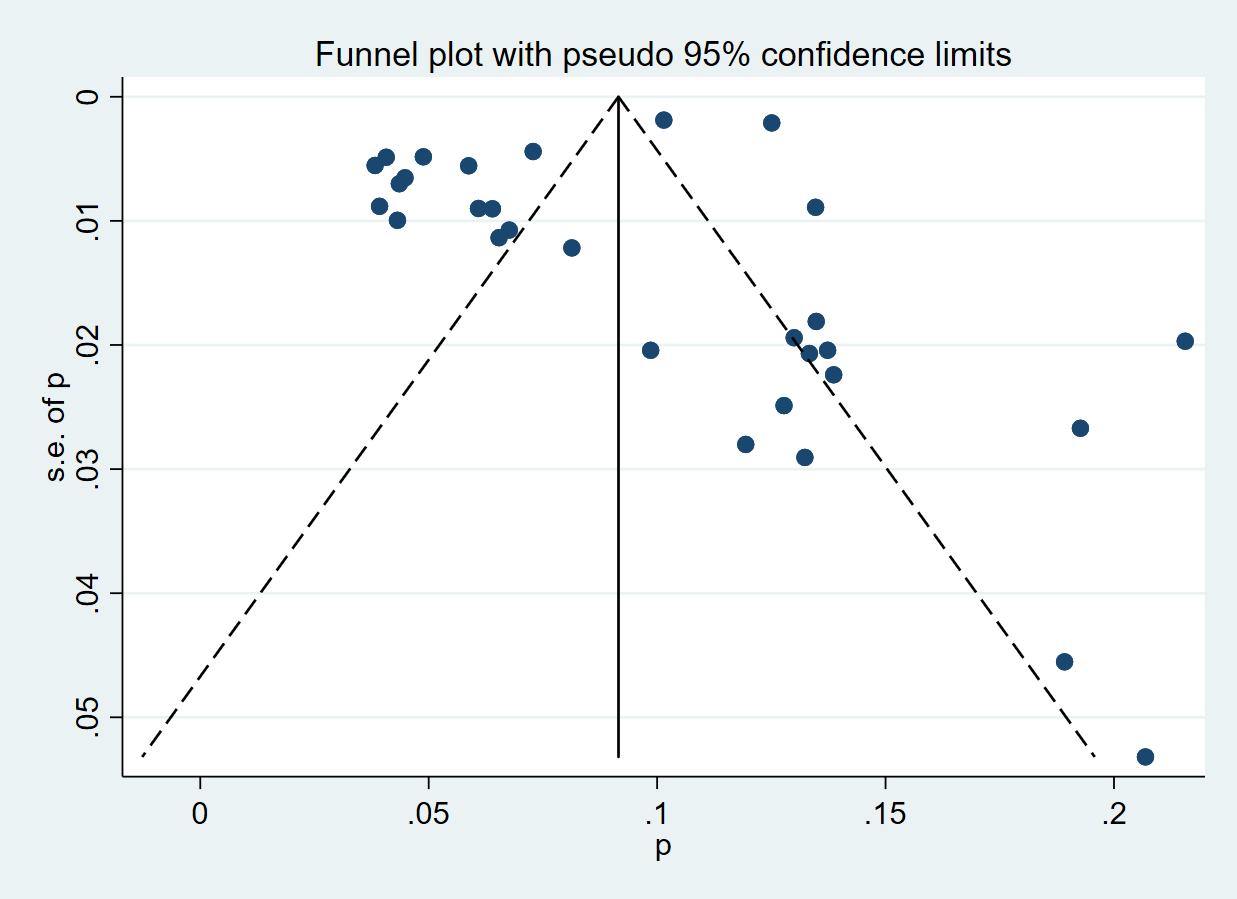


Figure S4. Funnel plot of postoperative ileus incidence


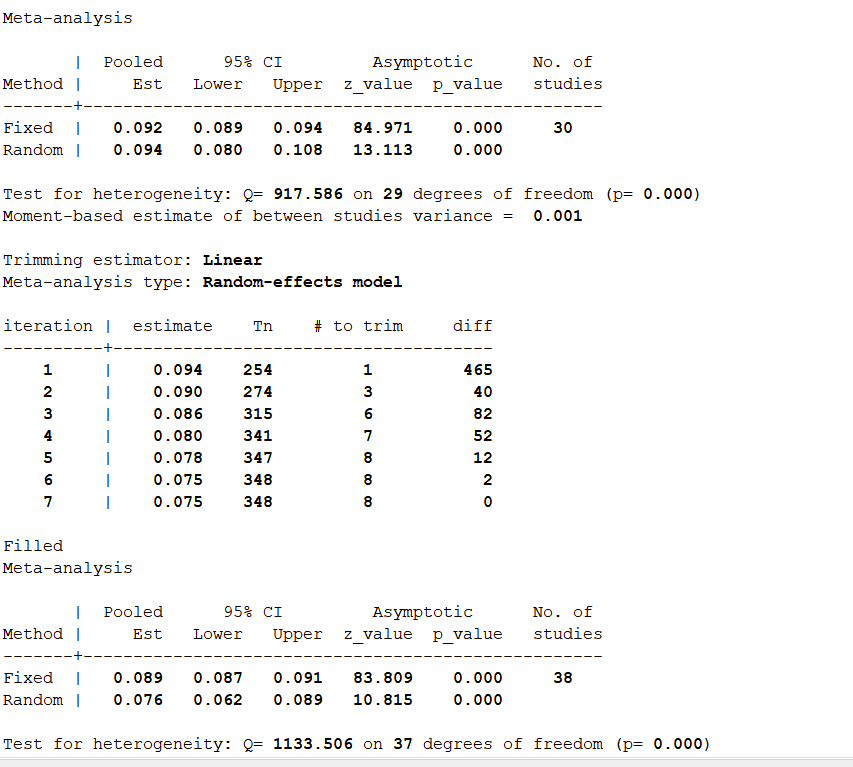


Figure S5. Trim-and-Fill analysis for the incidence of postoperative ileus


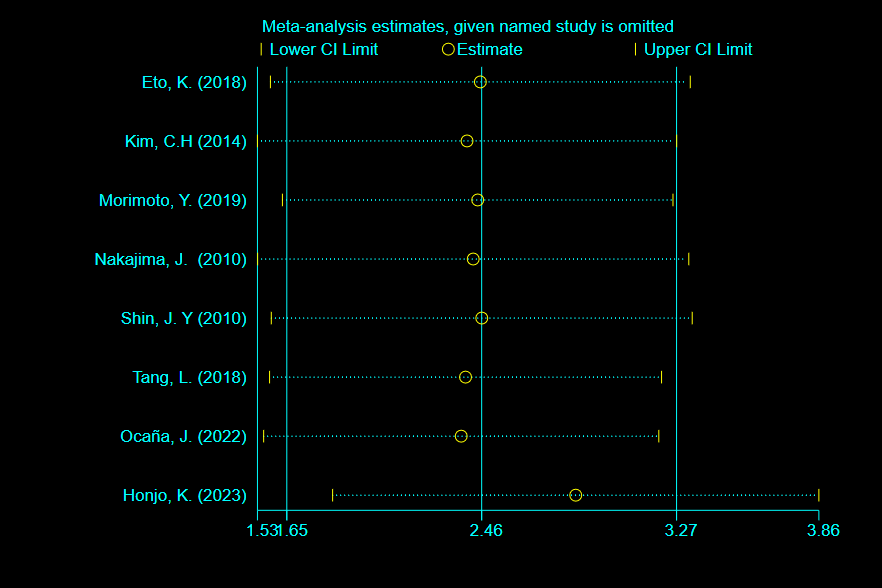


Figure S6. Sensitivity analysis of the meta-analysis of open surgery


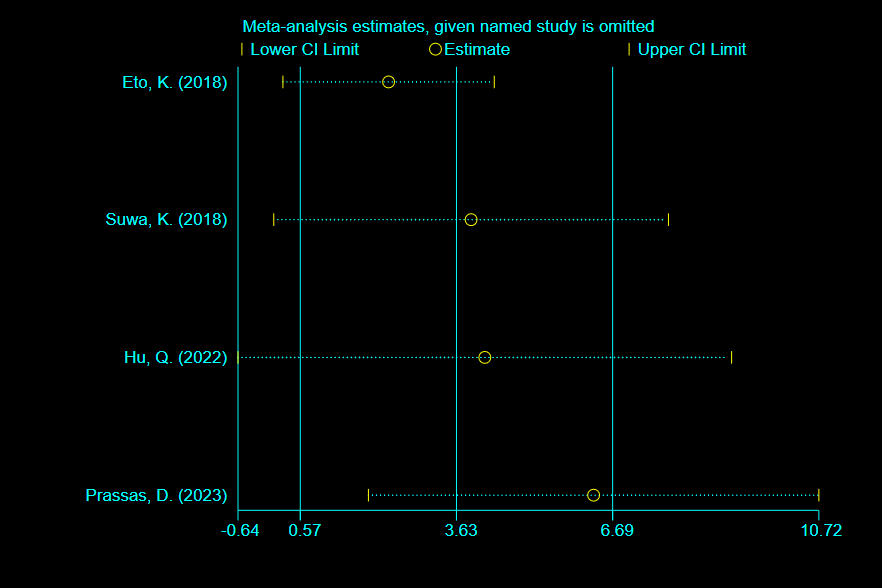


Figure S7. Sensitivity analysis of the meta-analysis of ileostomy


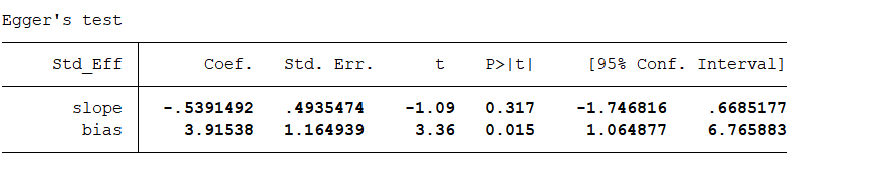


Figure S8. Egger's test of the meta-analysis of open surgery


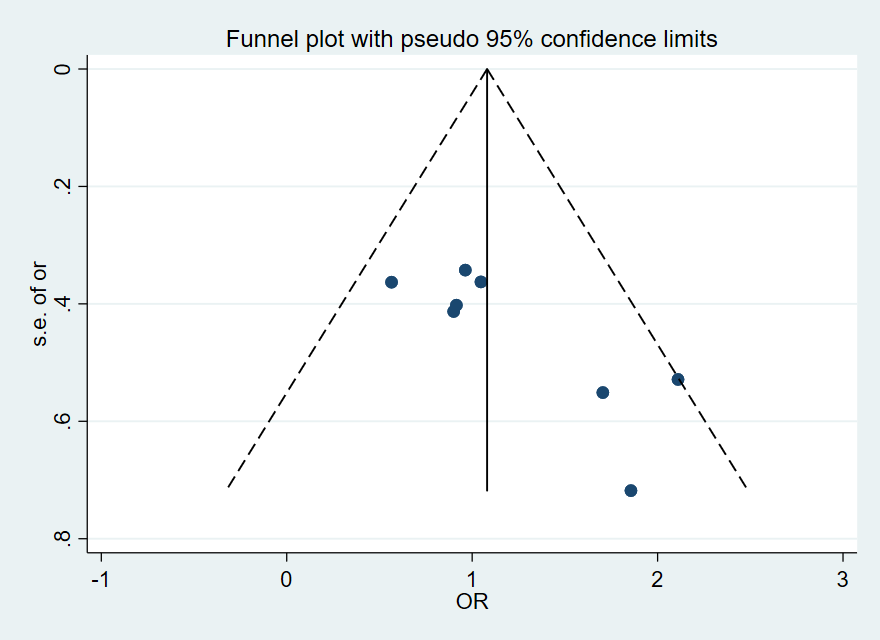
 Figure S9. Funnel plot of the meta-analysis of open surgery


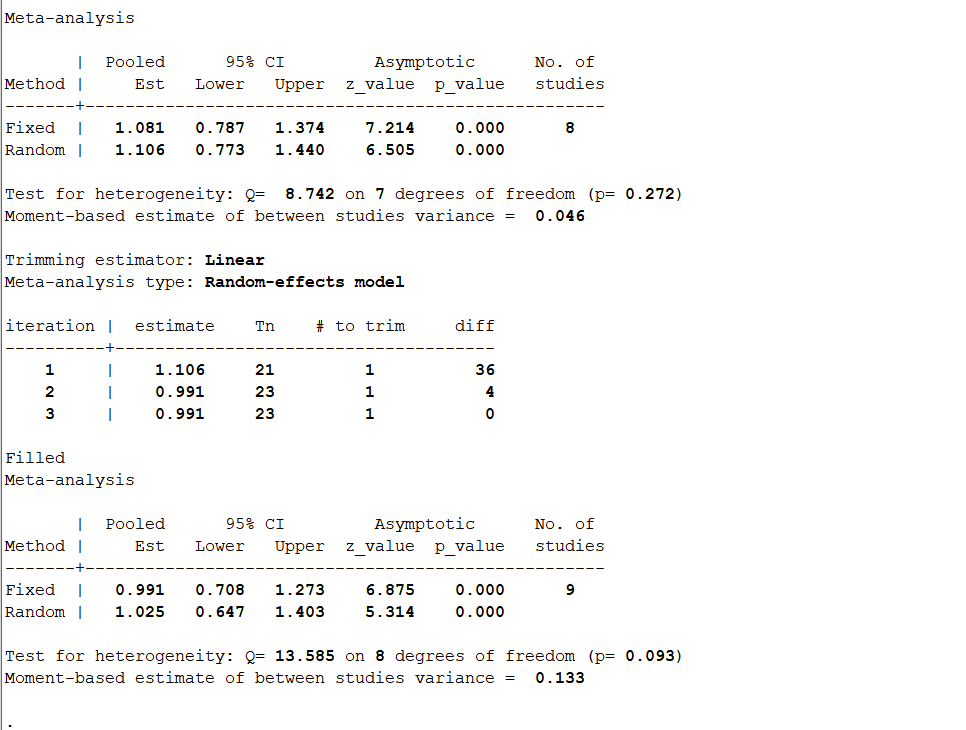


Figure S10. Trim-and-Fill analysis for open surgery


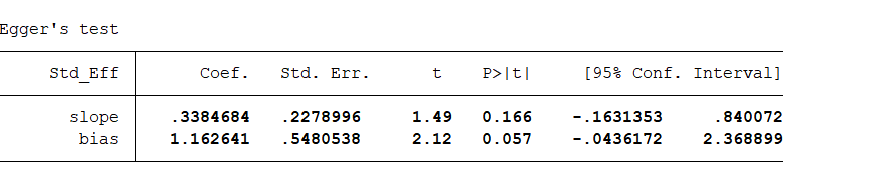


Figure S11. Egger's test of the meta-analysis of male


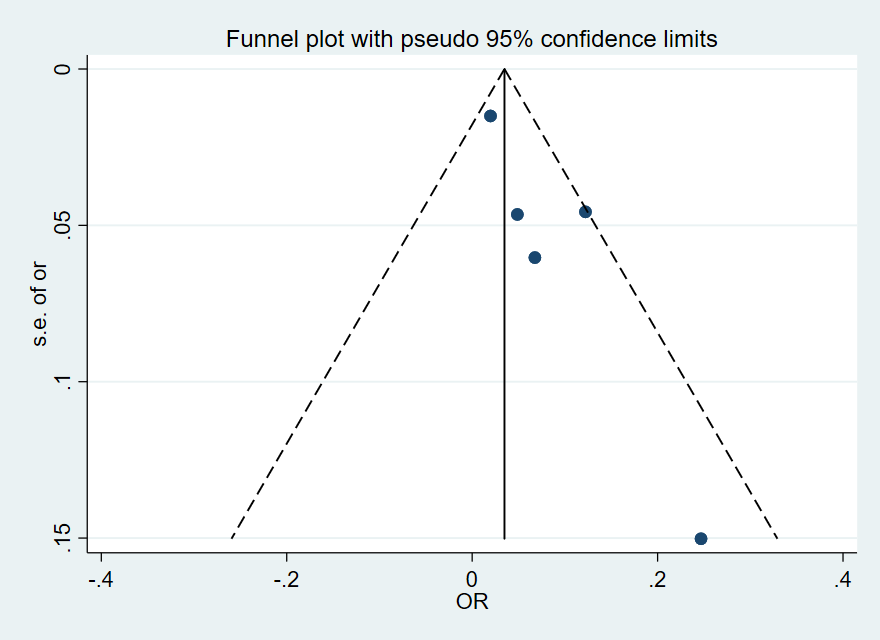


Figure S12. Funnel plot of the meta-analysis of male sex


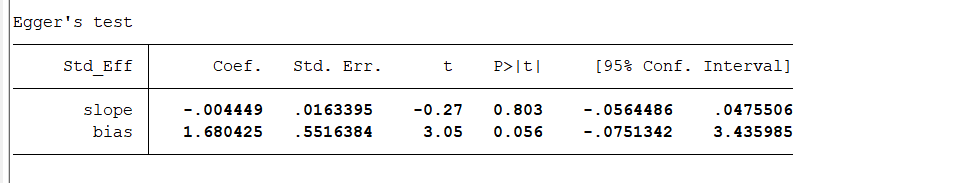


Figure S13. Egger's test of the meta-analysis of Age≥65 years


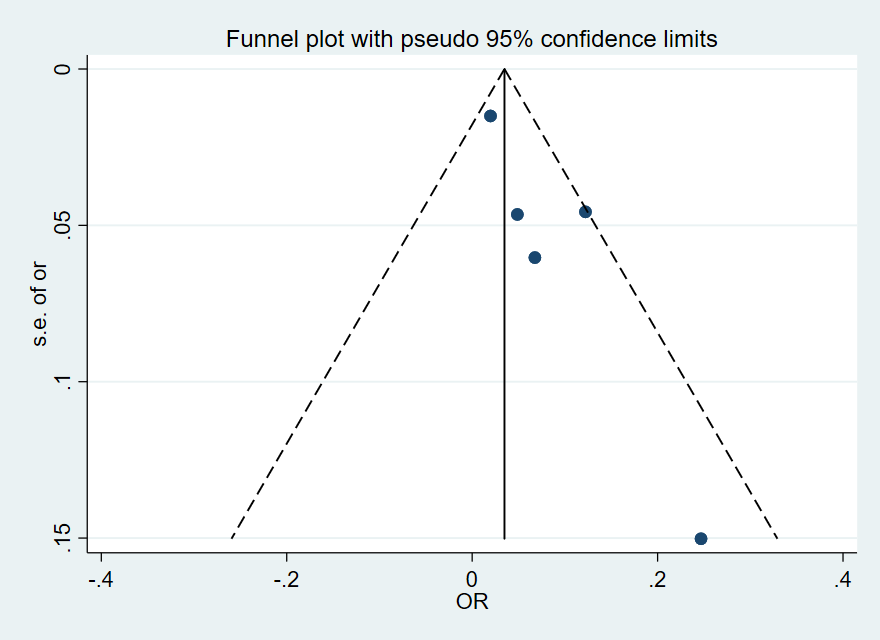


Figure S14. Funnel plot of the meta-analysis of Age≥65 years


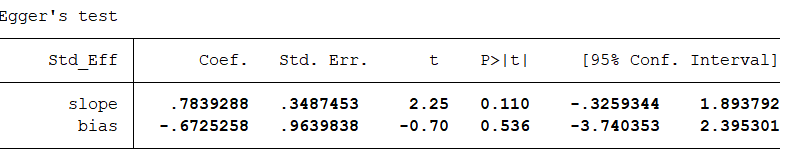


Figure S15. Egger's test of the meta-analysis of Operating time >3h


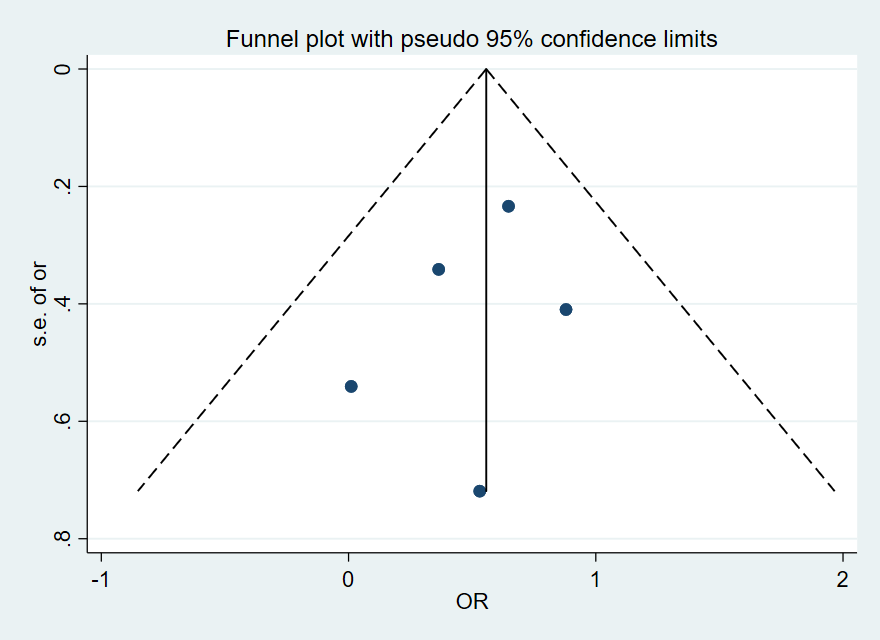


Figure S16. Funnel plot of the meta-analysis of operating time >3h


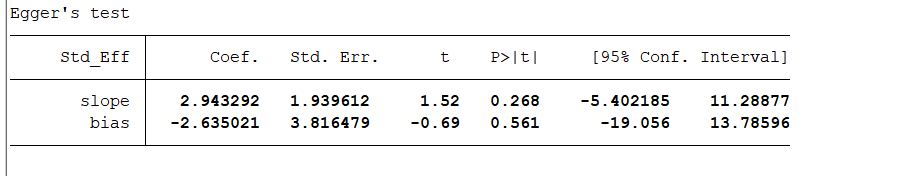


Figure S17. Egger's test of the meta-analysis of ileostomy


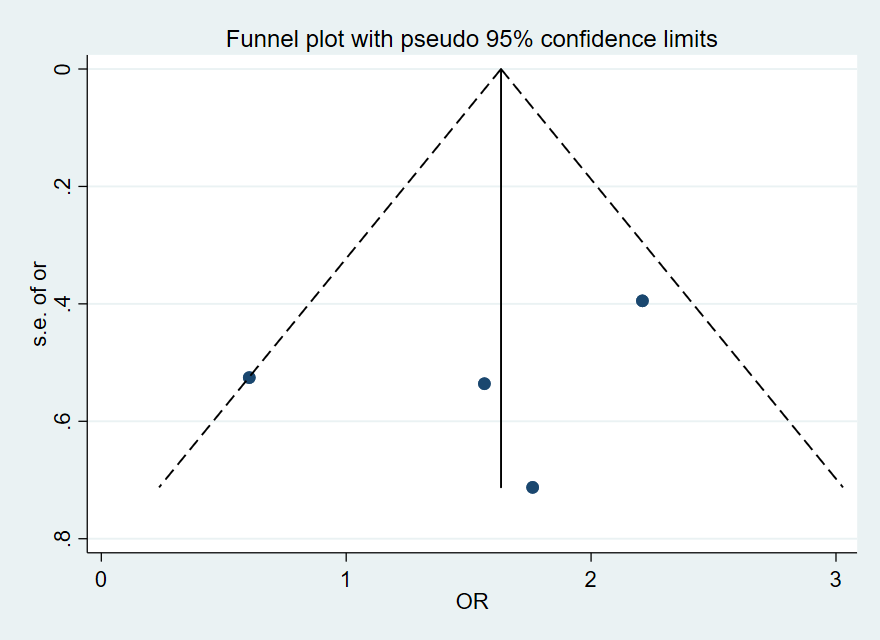
Figure S18. Funnel plot of the meta-analysis of ileostomy


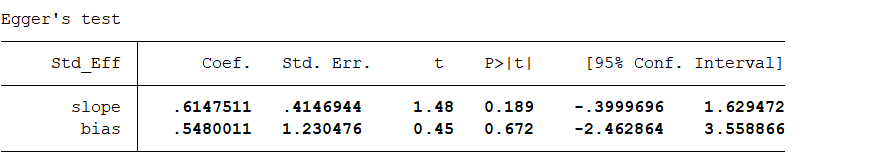


Figure S19. Egger's test of the meta-analysis of Previous abdominal surgery


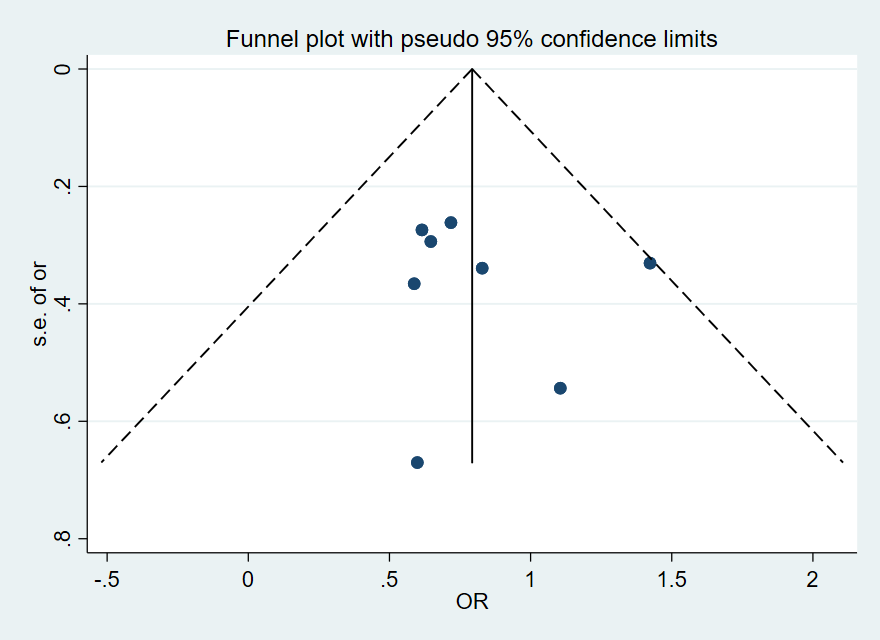


Figure S20. Funnel plot of the meta-analysis of Previous abdominal surgery
