# Supplementary material for: Prevalence and risk factors for postoperative ileus in colorectal cancer patients: a systematic review and meta-analysis
Source: Front Oncol. 2026 Jan 16;15:1742152. doi: 10.3389/fonc.2025.1742152 (PMC12855047; doi:10.3389/fonc.2025.1742152)
Supplement: Supplementary file 3 [file DataSheet3.doc]

**Table 1:** Covariates adjusted in multivariable analyses of included studies

| Study | Year | Adjusted covariates |
| --- | --- | --- |
| Campana, J. | 2017 | Age, Sex, Comorbidities, ASA grade, Tumor stage, operative time. |
| Eto, K. | 2018 | Sex, Open surgery, Ileostomy. |
| Garfinkle, R | 2020 | Age, Sex, BMI, ASA grade, Preoperative albumin, Preoperative bowel preparation, Operative approach, Operative time. |
| Grieco, M. | 2021 | CO2 pressure, Sex, Operative time. |
| Husarić, E. | 2016 | previous surgery, Operating time, Tumor-Node Metastasis, Postoperative complications. |
| Kim, C. H. | 2014 | Sex, Diverting stoma, Conversion to open surgery, Transfusion, Combined operation, Operation time. |
| Morimoto, Y. | 2019 | Sex, VO, Neoadjuvant chemotherapy, Open surgery, Operative time, Blood loss, Pelvic/intra‐abdominal abscess. |
| Nakajima, J. | 2010 | Sex, Open colectomy, Rectal cancer, Tumor size, operating time. |
| Nakamura, Y. | 2021 | Sex, Operative time, Blood loss, Repositioning the small intestine, Anastomotic leakage. |
| Rybakov, E. G. | 2017 | Age,Sex,BMI, Previous abdominal surgery, Moderate drinker vs Abstainer/light drinker, Heavier drinker vs Abstainer/light drinker, Opioid analgesics, Grade IV adhesions, Multivisceral resections, Haemotransfusion, Laparoscopic vs open, Blood loss. |
| Seo, G. H | 2018 | Age, Sex, Operation method, Cancer location, Institution volume. |
| Shin, J. Y. | 2008 | ASA grade, Previous laparotomy, Local remnant tumor. |
| Shin, J. Y | 2010 | Age, Previous laparotomy, Operative type, Open surgery, No treatment with antiadhesive. |
| Suwa, K. | 2018 | Sex, Previous abdominal surgery, Low tumor, Low anterior resection, Ileostomy, Operation time, D3 lymph node dissection. |
| Tang, L. | 2018 | Surgical approach, Radiotherapy, Antiadhesive materials, Pelvic peritoneum sutured, Blood loss, Tumor stage (TNM). |
| Namba, Y. | 2021 | Sex, Performance status, Intraoperative in–out balance per body weight. |
| Hu, Q. | 2022 | Sex, Neoadjuvant chemoradiotherapy, Location (Rb), Temporary diverting ileostomy, Operation time. |
| Matsui, R. | 2022 | Location Right colon, Preoperative chemotherapy, Preoperative antithrombotic drug use, Epidural anesthesia, Pathological nerve infiltration, Postoperative complication Clavien-Dindo≥3. |
| Nakamura, Y. | 2022 | Sex, Operative time, blood loss, Repositioning the small intestine, Anastomotic leakage. |
| Ocaña, J. | 2022 | Age, Sex, ASA grade, Previous abdominal surgeries, Index surgery approach, Modality of neoajuvant treatment, PO complication at index surgery, Postoperative ileus at index surgery, Adjuvant chemotherapy, Chemotherapy, Mucositis, Physiological stimulation. |
| Sasaki, M. | 2022 | BMI, Laparoscopy, blood loss, Stoma, SMI. |
| Fujiyoshi, S. | 2023 | Bleeding, COPD, BMI, Sex. |
| Honjo, K. | 2023 | Tumor location (rectum), Anastomotic leakage, Previous abdominal surgery, Operating time, Operative procedure, Blood loss, Use of APM. |
| Prassas, D. | 2023 | Age, Previous abdominal surgery, Ileostomy. |
| Uchida, F. | 2023 | Sex, Comorbidities, Previous abdominal surgery, Stent insertion, Tumor stage. |
| Yanagisawa, T. | 2023 | Age, operative time, stoma, anastomotic leakage |
| Cai, W. T. | 2024 | Age, BMI, Primary site, Neoadjuvant chemotherapy, CA19-9, CA72-4. |
| Emile, S. H. | 2024 | Age, Emergent surgery, Extended colectomy, Green GIA for anastomos, Operation time. |
| Yehaiya, M. | 2024 | Tumor stage, Preoperative hypoproteinemia, Previous abdominal surgery, Preoperative intestinal obstruction, Lymph node metastasis. |
| Kim, S. | 2025 | Age, Sex, Anastomosis type |
